# Alpha oscillations and traveling waves: Signatures of predictive coding?

**DOI:** 10.1371/journal.pbio.3000487

**Published:** 2019-10-03

**Authors:** Andrea Alamia, Rufin VanRullen

**Affiliations:** Centre de Recherche Cerveau et Cognition (CerCo), CNRS, Université de Toulouse, Toulouse, France; Yeshiva University Albert Einstein College of Medicine, UNITED STATES

## Abstract

Predictive coding is a key mechanism to understand the computational processes underlying brain functioning: in a hierarchical network, higher levels predict the activity of lower levels, and the unexplained residuals (i.e., prediction errors) are passed back to higher layers. Because of its recursive nature, we wondered whether predictive coding could be related to brain oscillatory dynamics. First, we show that a simple 2-level predictive coding model of visual cortex, with physiological communication delays between levels, naturally gives rise to alpha-band rhythms, similar to experimental observations. Then, we demonstrate that a multilevel version of the same model can explain the occurrence of oscillatory traveling waves across levels, both forward (during visual stimulation) and backward (during rest). Remarkably, the predictions of our model are matched by the analysis of 2 independent electroencephalography (EEG) datasets, in which we observed oscillatory traveling waves in both directions.

## Abstract 2

A predictive coding model explains the spatio-temporal dynamics of alpha oscillations recorded in human brain experiments, including traveling waves whose direction of propagation depends on the cognitive state.

## Introduction

**Fig 1:**
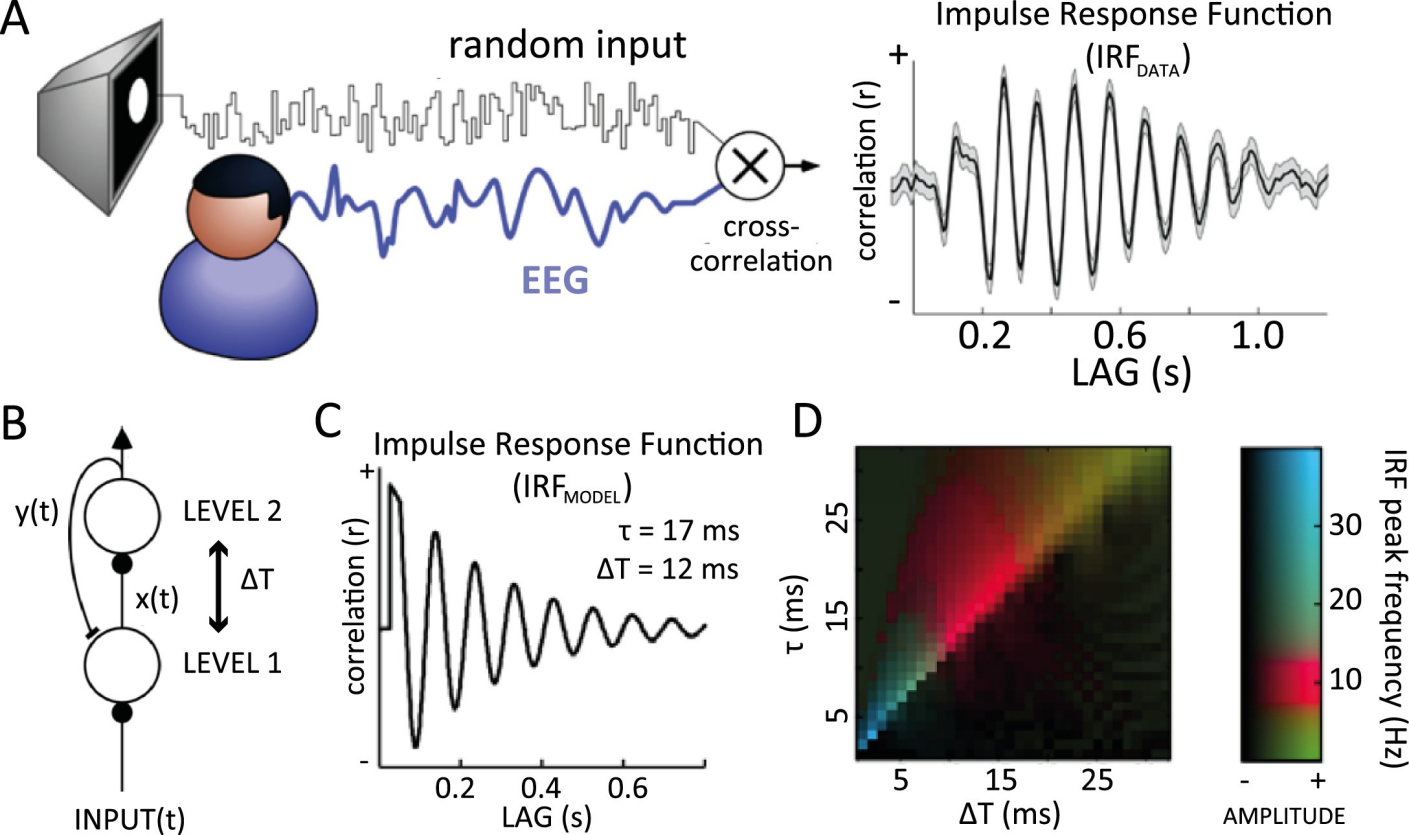
Alpha-band oscillations in human IRF and in a predictive coding model. (A) Cross-correlating the white-noise sequence of a stimulus with simultaneously recorded EEG produces an IRF, which reverberates at 10 Hz for several successive cycles (1 representative subject shown here, electrode POz). (B) A simple predictive coding model in which the hierarchically higher level makes predictions y(t) about the input received by the lower level, and the residual x(t) (prediction error) is used to update the next prediction. Residual and prediction signals are transmitted to the next or previous level (respectively) with a communication delay ΔT. Such a model, with physiologically plausible parameters, generates an oscillatory IRF at 10 Hz. (C) The oscillatory IRF produced by the model, with communication delay ΔT = 12 ms, and neural membrane time constant τ = 17 ms. (D) Systematic exploration of these two parameters suggests that alpha reverberation is a robust phenomenon (red colors) within a biologically plausible range of values. EEG, electroencephalography; IRF, impulse response function.

**Fig 2:**
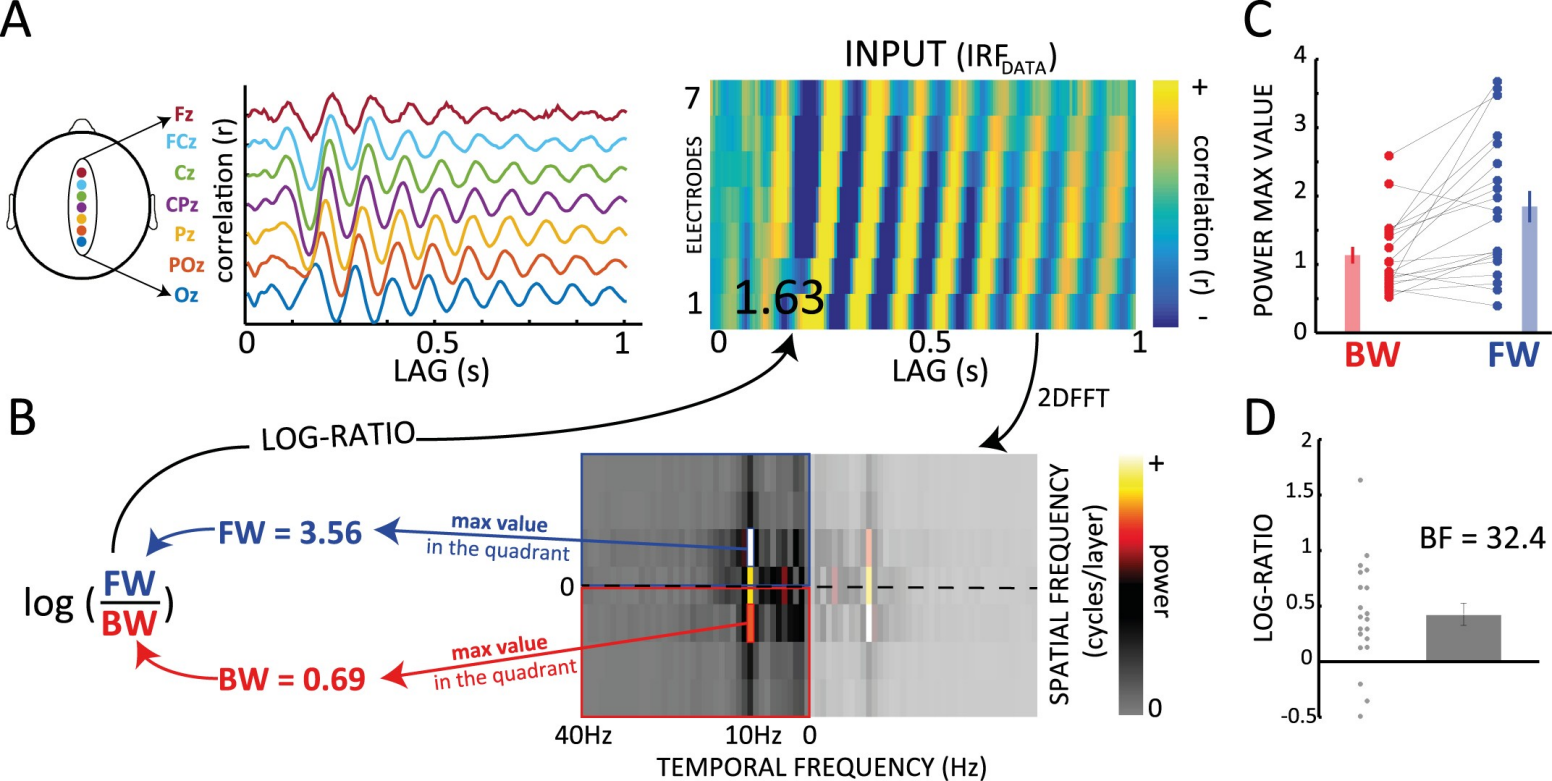
Alpha-band traveling waves in EEG IRFs. (A-B) IRF as traveling waves can be observed over the 7 midline electrodes of the 10–20 system (Oz to Fz; 1 representative subject). From the 2D map obtained by stacking signals from the 7 electrodes, we compute a 2D FFT and derive a log ratio between spectral quadrants that quantifies the direction of the waves. Positive values are associated with FW waves and negative values with BW ones. (C) BW (red) and FW (blue) values (max value in the corresponding quadrant of the 2D FFT) computed over 20 subjects. Participants were watching a white-noise luminance sequence (see Fig 1A). (D) Log ratios computed from the values in (C). The BF of a 1-sample *t* test against zero confirms the presence of FW waves. See S1 Data. BF, Bayes factor; BW, backward; EEG, electroencephalography; FFT, fast-Fourier transform; FW, forward; IRF, impulse response function.

Predictive coding is a popular computational paradigm to model sensory information processing in the brain, and it has been proposed to explain several cognitive and physiological observations [1]. Could it also explain the emergence of alpha-band oscillations and some of their distinguished features (e.g., traveling waves)? Alpha rhythms (8–12 Hz) are the most predominant oscillations in the human brain, even though their functional role remains controversial. On the one hand, alpha is generally stronger in the absence of visual inputs, or when visual inputs are actively ignored—hence, a proposed inhibitory role for alpha rhythms [2–6]. On the other hand, alpha has been suggested to contribute to the temporal framing of sensory inputs [7,8], and it can sometimes be positively correlated with visual inputs. For example, a recent study reported strong and long-lasting (up to approximately 1 s) alpha-band oscillations in the visual impulse response function (IRF; [9]). The IRF is computed by cross-correlating occipital electroencephalography (EEG) signals recorded from human subjects watching a dynamic sequence of random (white-noise) luminance values with the corresponding stimulus sequence (Fig 1A). It is, therefore, a direct reflection of visual sensory processing. The existence of significant input–output correlations at lags of nearly 1 s is surprising, given the typical neural time constants (<50 ms) and the short-lived nature of visual-evoked responses (<0.5 s). Additionally, a recent study from our group [10] showed that alpha IRF oscillations propagate as a traveling wave across the cortex in an occipital-to-frontal direction (Fig 2A and 2C). This finding is in line with other recent intracranial studies about alpha-frequency cortical traveling waves [11–14]. Yet, the mechanisms underlying such oscillations remain debated. Could it be possible that a common computational scheme, predictive coding, gives rise to alpha-band oscillations and their typical traveling-wave dynamics?

Here, we address this question in 2 steps. First, we demonstrate the presence of similar long-lasting alpha-band oscillations in the IRF of a simple 2-level model of visual cortex, simulating, e.g., 2 neighboring brain regions. Our model does not explicitly integrate or maintain information over extended periods but merely tries to predict what comes next based on what was just there, i.e., predictive coding. The key insight is that typical neural communication delays between cortical areas can give rise to a reverberation of visual inputs at alpha frequency, as observed experimentally. Second, we expand our predictive coding model by increasing the number of hierarchical levels to explore the occurrence of alpha-band traveling waves in a hierarchical network. By “traveling waves,” we mean the propagation of neural signals through contiguous brain regions; however, we also insist on the rhythmic nature of these waves, i.e., not a single propagating wave front but a periodic succession of such wave fronts—hence, “alpha-band traveling waves.” After having observed that this hierarchical model produces (1) forward (FW) traveling waves during sensory processing and (2) backward (BW) traveling waves in the absence of sensory input, we turned to experimental EEG data to verify these predictions. Remarkably, we found FW waves in human EEG when participants were attending to visual stimulation and BW waves when participants' eyes remained closed, in agreement with our model’s predictions.

Importantly, our model considers interactions between cortical (and possibly subcortical) regions, rather than the detailed laminar patterns of connections and neuronal populations within each brain region. It is thus not intended as a precise model of alpha-rhythm generation in the brain, nor is it expected to explain every known experimental property of alpha oscillations. Rather, we explore the consequences of implementing the predictive coding algorithm within biological substrate with known temporal constraints; we find emerging spatiotemporal dynamics that match electrophysiological observations made in similar experimental situations: alpha-band oscillations, echoes, and bidirectional traveling waves. As for any computational model, this agreement between model and experimental observations could, of course, be fortuitous; we speculate, however, that it may reflect a relationship between predictive coding and alpha rhythms in the brain.

## Results

### Two-level model and alpha-band oscillations

In an attempt to explore the emergence of alpha-band IRF oscillations during the visual processing of white-noise luminance sequences (Fig 1A), we initially implemented a simple model of 2 hierarchical levels. The model architecture was inspired by the classic predictive coding model of Rao and Ballard (1999) [1], in which each level attempts to “explain away” (via inhibitory feedback) the activity pattern in the previous one, which only communicates the “unpredicted” residual signals (via feedforward excitation). Since, in our case, the stimuli are strictly temporal luminance sequences, without any meaningful spatial arrangement (Fig 1A), the original model could be simplified greatly by ignoring spatial selectivity and considering a single population of neurons in each of 2 connected areas (corresponding, e.g., to lateral geniculate nucleus [LGN] and V1 of the primate brain). The resulting circuit is illustrated in Fig 1B. The specificity of the present approach is to consider the effects of the communication delay ΔT between the two levels (assumed here to be symmetric, for simplicity). The population in the first level encodes the residual between the input sequence and the "prediction" received, with a delay ΔT, from the second level. The instantaneous response *y(t)* of the population in this second level is governed by a differential equation (see Materials and methods) composed of 2 terms: the first term determines the integration of inputs from the first level, with a delay ΔT, and the second is a decay term ensuring that neurons eventually return to their resting state in the absence of inputs. The temporal dynamics of neuronal integration and decay are governed by 2 time constants, respectively τ and τ_D_, the latter of which was fixed to τ_D_ = 200 ms. We computed the model’s IRF by cross-correlating each random input luminance sequence *input(t)* with the corresponding output *y(t)* of the second level and averaging the result over 200 trials. That is, we assumed here that the activity of the hierarchically higher level (prediction) can be used as an approximation of the EEG signal (generally, in predictive coding, prediction error signals are associated with superficial pyramidal cells that are the predominant contributor to EEG; see [68]; however, because of our simplified modeling of the predictive coding scheme, the fluctuations in prediction unit signals and prediction errors are highly correlated. This licenses us to treat the prediction signals as a summary of neuronal message passing; however, similar results with only a phase difference were found using activity of the first level instead). With biologically plausible values for the τ and ΔT parameters (respectively 17 ms and 12 ms), the model’s IRF oscillated at a frequency within the alpha range, thus qualitatively replicating the experimental observations (Fig 1C).

### Sensitivity to parameters

We investigated the dependence of IRF oscillations (and of their frequency) on the model’s 2 free parameters τ and ΔT. First, we employed numerical simulations, similar to the one described above (Fig 1C). A fast-Fourier transform (FFT) applied on the simulated IRF was used to measure the peak oscillatory amplitude and its corresponding frequency. These two measures are color-coded in Fig 1D, displaying the results of a systematic exploration of parameter space. Several combinations of parameters gave rise to oscillatory IRFs (brighter colors); among these, IRF oscillations in the alpha (8–13 Hz) frequency range were particularly frequent (red colors). In particular, alpha-band IRF oscillations systematically arose when τ and ΔT lay in their “biologically plausible” range of respectively 15–25 ms [15–18] and 10–15 ms [19–21].

Second, we also derived an analytical solution to a simplified version of our differential equation system (see Materials and methods). The solution revealed that (1) IRF oscillations occur mainly when the parameter τ lies slightly above ΔT (the optimal oscillatory situation corresponding to *τ* ≈ 1.27 Δ*T*) and (2) when oscillations occur, the oscillation period equals 8 × ΔT. In other words, alpha-range oscillations (8–13 Hz) correspond to ΔT values between 10 and 15 ms and τ values around 15–20 ms. Therefore, both of these analytical conclusions directly confirm the results of the numerical simulations.

### Multilevel model and traveling waves

#### FW traveling waves during visual processing

**Fig 3:**
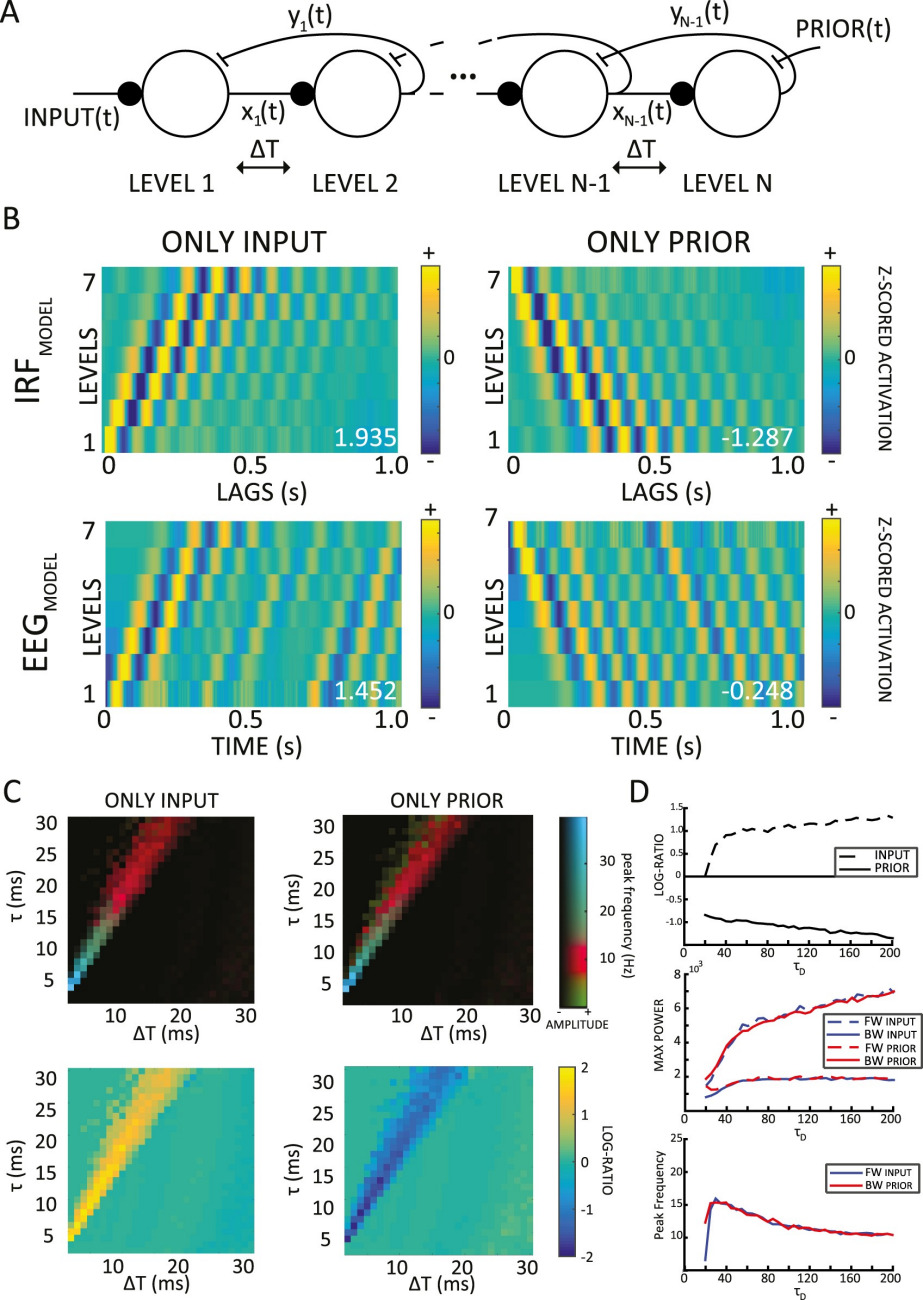
Alpha-band traveling waves in a hierarchical predictive coding model. (A) Multilevel version of the model: the same parameters (ΔT = 12 ms and τ = 20 ms) are used throughout. The model is fed either a time-varying input (left) or a time-varying prior signal (right), reflecting top-down expectations computed in other parts of the brain. (B) Two-dimensional maps with only input (left column) or prior signals (right column): traveling waves are visible both in the IRF (computed by cross-correlating prediction signals with input or prior signals, top row) and in the raw prediction signals (considered as a proxy for the EEG, lower row). All the values have been z-scored level-wise for visualization purposes only. The numbers in white show the log ratio for each simulation: positive or negative numbers reveal respectively FW or BW waves. (C) Systematic exploration of parameters ΔT and τ (with τ_D_ kept constant at 200 ms). The upper panels show the temporal frequency of the peak spectral component in the 2D Fourier spectrum, with the same color scheme as in Fig 1D. In the lower panels, the direction of traveling waves was similarly explored (color indicates log ratio, with absolute values below 0.2 set to 0 for increased readability). When the model was fed the input or the prior, we observed mostly positive or negative values, respectively. (D) Explorations of model behavior as parameter τ_D_ is varied systematically (x-axis, from 20 ms to 200 ms, stride of 5). From top to bottom, the panels represent log ratios, max spectral power values, and temporal frequency of the max spectral value from the 2D FFT. Each value is an average over 20 simulations (with ΔT and τ kept constant at 12 and 20 ms, respectively). The code of the model and the simulation is available at https://github.com/artipago/echoPred. BW, backward; EEG, electroencephalography; FFT, fast-Fourier transform; FW, forward; IRF, impulse response function.

After having explored the emergence of alpha oscillations in a simple predictive coding model, we investigated whether our model could reproduce other features of alpha-band oscillations, such as their traveling-wave dynamics. Consequently, we extended the model to a multilevel version (Fig 3A), in which τ_D_ (decay time constant) remained fixed at 200 ms, and ΔT and τ were respectively 12 ms and 20 ms in all simulations. We chose these values based on the results of the previous parameter-space exploration, considering that these parameters are both biologically plausible, and produced IRF alpha oscillations in a 2-level version of the predictive coding model (for a detailed investigation of the parameter space of the multilevel model, see below and S4 Fig and S5 Fig). As previously, the prediction signals from each level in our model were treated as the equivalent of EEG signals from distinct electrodes over the human brain, and we used 7 levels to facilitate comparison with experimental data (Fig 2A). Nonetheless, qualitatively similar results were obtained using lower or higher electrode numbers. We created 2D maps by stacking the EEG or IRF signals (x-axis) from the 7 levels (y-axis, see Fig 2B). To quantify the presence and direction of waves, we then extracted the maximum values in the upper and lower quadrants of the 2D FFT of these maps, representing respectively the amount of FW and BW signal propagation across levels (Fig 2A and 2B). Finally, the log ratio of these two values quantified the overall direction of the waves: positive ratios indicate predominantly FW waves, whereas negative ratios reveal mostly BW waves. In a first simulation, comparable to the experimental setup described in Fig 1A, we presented the multilevel model with white-noise inputs only. As shown in the left column of Fig 3B, the model response to these inputs displayed FW alpha-band oscillatory traveling waves, proceeding from lower to higher levels; this oscillatory propagation was visible when we applied our wave quantification method both to IRF signals (after cross-correlation with the stimulus sequence) and to the raw EEG signals. In other words, instead of looking for systematic differences in the phase of alpha oscillations (over channels) in the IRFs, we tested for the same effects in the raw EEG signal. Whereas on the one hand, the presence of FW waves in the model’s IRF (Fig 3B, top left panel) was the anticipated model outcome (see [10] but also Fig 2A), on the other hand the presence of traveling waves in the raw (simulated) EEG signal was not readily predictable. Nonetheless, we observed a clear FW propagation in the raw EEG signals of our model, as shown in the bottom-left panel of Fig 3B, suggesting that oscillatory traveling waves during sensory processing could be directly visible in EEG recordings (without stimulus cross-correlation). This prediction will be verified in one of the following sections.

#### BW waves in the absence of inputs

Next, we further expanded the model functionality and introduced an endogenous signal at the last level, namely the prior *y*_*N + 1*_*(t)* (Fig 3A, where *N* is the number of levels). This signal is functionally equivalent to the prediction *y*_*L*_*(t)* generated by the model at all previous levels 1 to *N*, but it is assumed here to arise from higher-level brain regions that are not part of the model and thus to influence the activity of the last level as a top-down prediction. In order to investigate the consequences of this new “prior” signal, we first set the model’s input to zero and the prior as a time-varying white-noise signal, allowing us to compute a prior-driven IRF at each level. Note that these design choices were only made to facilitate computations and comparison of input-driven and prior-driven IRFs and do not reflect any assumption about the statistical structure of inputs or priors under natural conditions of stimulation (natural inputs are not random and typically do not display a flat power spectrum; similarly, top-down brain signals are unlikely to have white-noise properties). Contrary to the waves’ direction in the previous simulation, the activity of the prior in the absence of inputs generated an oscillatory BW alpha-band traveling wave, which propagated from higher to lower levels (Fig 3B, top right panel). As previously, we also investigated whether the raw simulated EEG signals would exhibit similar dynamics: as shown in the lower-right panel of Fig 3B, we found that this was indeed the case, observing EEG traveling waves proceeding from higher to lower levels. All in all, these results indicate that—at least in the model—EEG recordings are sufficient to identify traveling waves propagating in both directions.

In a final simulation, the model was fed with both the input and the prior as two independent white-noise signals. In this case, the dynamics induced by the inputs appeared to dominate, which was mathematically expected given τ_D_ >> τ. Consequently, the model revealed predominantly FW waves in this simulation (see S1 Fig for an overview of all possible scenarios and S5 Fig for explorations of network dynamics with different values of parameter τ_D_).

All in all, the chief conclusion of these simulations is that hierarchical predictive coding gives rise to oscillatory traveling waves that can be seen in both the EEG and IRF signals. Sensory inputs generate FW waves, whereas top-down priors induce BW waves. Although the presence of alpha-frequency FW waves in the IRF during visual processing had been previously demonstrated ([10], see also Fig 2A and 2C), our model makes further predictions that have not been experimentally validated yet. In the following sections, we first investigate the robustness of these results by varying the model free parameters and then test whether these new predictions are matched by experimental EEG data.

#### Multilevel model parameter space analysis

As in the 2-level version of the model, we investigated how the traveling-wave dynamics change upon varying the model’s free parameters τ and ΔT in the presence of either input or top-down prior signals (see S4 Fig and S5 Fig for all cases, including combined stimulation with inputs and top-down priors). For completeness, we also explored (separately) the influence of parameter τ_D,_. In a first analysis, we investigated the model dynamics by varying τ and ΔT while keeping τ_D_ constant (as previously, τ_D_ = 200 ms). As in the 2-level analysis, the systematic exploration of oscillatory frequency in the multilevel model (measured as the temporal frequency of the peak spectral power in the 2D Fourier space) confirmed the presence of alpha-band oscillations in the physiological range (Fig 3C, top), i.e., 10 ms < ΔT < 15 ms and 15 ms < τ < 25 ms. This analysis demonstrates that shorter time constants and temporal delays (i.e., both τ and ΔT < 10 ms) can produce faster oscillations and waves (i.e., in the beta and gamma range), which may be more compatible with the temporal dynamics of other sensory domains, e.g., the auditory system. Regarding the log ratios, we observed positive or negative values, respectively, when either the input or the prior signals were fed to the model (Fig 3C, bottom). The traveling waves (positive or negative log ratios) typically occurred in the regime that was strongly oscillatory (bright colors in the upper plots).

Furthermore, it is important to point out that the membrane time constant term τ in our equations, which governs the rate of integration of the residuals into a neuron’s prediction, is not equivalent to the network time constant of signal propagation in the cortex. Murray and colleagues demonstrated that the latter, measured as the time constant of the autocorrelation of neural signals, spans over few hundreds of milliseconds [22] and increases as a function of the hierarchical level. Remarkably, in our model, the autocorrelation of each level’s prediction yields a time constant matching these experimental observations: as shown in S6 Fig, these network time constants—computed by fitting the positive side of the autocorrelation function with a decreasing exponential—range between 100 and 500 ms and increase as a function of the level, in agreement with the experimental recordings [22]. This global behavior emerges despite each hierarchical level using the same integration time constant τ = 12 ms and the same decay time constant τ_D_ = 200 ms.

In a second analysis (Fig 3D), we kept both τ and ΔT constant (τ = 20 ms, ΔT = 12 ms), whereas τ_D_ varied from 20 to 200 ms (stride equal to 5). We did not test values lower than 20 ms, as τ_D_ < τ would imply a faster timescale for hierarchically higher processes than for lower-level ones, which is theoretically [23] and experimentally [22,24–26] implausible. In each simulation, we computed the log ratio, the maximum power value of the 2D FFT and its corresponding frequency, for both FW and BW waves (i.e., separately for opposite quadrants of the 2D Fourier space), averaging over 20 simulations. Results show that the model behavior is very robust to changes in τ_D_, at least for most of the explored range (τ_D_ > 50 ms). As τ_D_ decreases and approaches τ (approximately 20 ms), the log ratios progressively converge to zero while the max power values reach baseline levels; i.e., the waves tend to disappear (at the same time, their frequency increases slightly to about 15 Hz; Fig 3D).

Furthermore, as the model is fully linear (for simplicity), in a separate analysis we also investigated the effect of adding a nonlinearity. Specifically, we introduced a sigmoid function at each level, and we scaled the input and the prior accordingly. Importantly, we observed a similar pattern of results in each simulation (see S8 Fig), with FW and BW waves in both the IRF and the raw EEG when the model was fed with only the input or the prior, respectively.

### Traveling waves in human EEG data

Our simulations show that hierarchical predictive coding can produce oscillatory traveling waves propagating both feedforward and BW. The model predicts that input processing produces mostly feedforward waves, whereas BW waves are generated by higher-level endogenous signals and most visible in the absence of visual inputs. Finally, the model suggests that even though cross-correlation and IRFs may be helpful to reveal the traveling waves, their dynamics could also be directly visible in the raw EEG signals. To test these predictions, we turned to real human EEG data to investigate (1) whether we could observe traveling waves in both IRF and EEG signals, as predicted by our model, and (2) whether their direction is task-dependent (e.g., sensory processing versus rest).

#### FW traveling waves during visual processing

**Fig 4:**
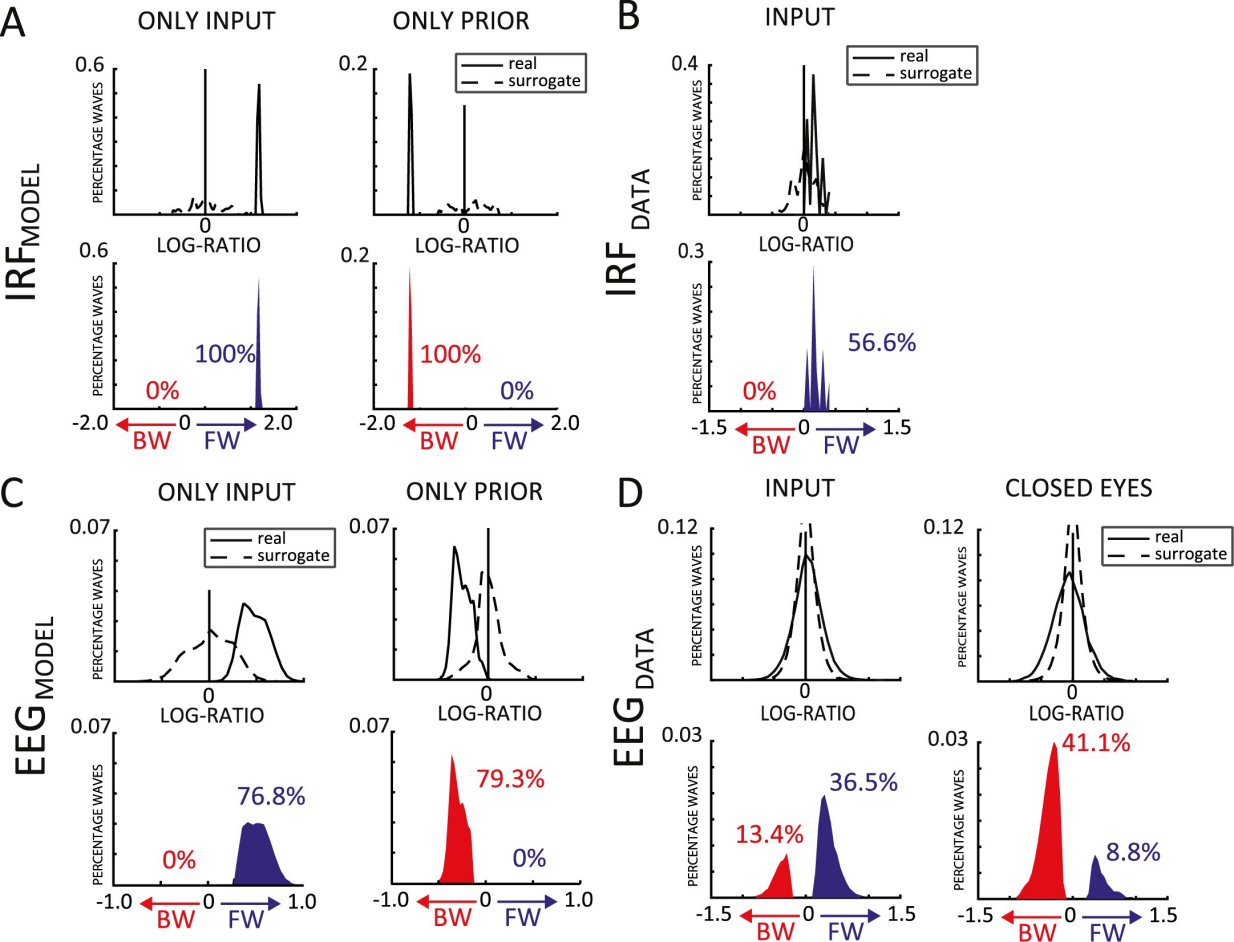
Quantification of traveling waves in human EEG data and in model simulations. (A) The first row shows the real (solid line) and shuffled (dashed line) distributions of log ratios computed over the IRF obtained during 2 sets of simulations, cross-correlating prediction signals (a proxy for the EEG in our model) either with the sensory inputs (first column), or with the top-down prior signals (second column). The second row shows the difference between the two, focusing on positive values, i.e., the traveling-wave events in the data that occur more often than predicted by the null distribution. The proportion of significant BW and FW waves are shown in red and blue, respectively. (B) Same as (A), but for the IRF computed in the INPUT human EEG dataset (during human EEG experiments, we only have direct access to the visual input signals, but the top-down priors, if any, remain unknown and cannot be used for cross-correlation). (C,D) Same as (A,B), but based on log ratio values computed directly in 1s EEG epochs, rather than in the IRF (for the model, we use the prediction signals y_i_ as a proxy for the EEG signals). Experimental human EEG results (B,D) follow the same qualitative pattern as the simulations (A,C). All data are available at: https://osf.io/nc4rg/?view_only=bb06fe996d1f49b285dc25464eec7ac7. BW, backward; EEG, electroencephalography; FW, forward; IRF, impulse response function.

Our group recently showed that alpha-band IRFs can be interpreted as FW traveling waves during visual perception [10]. We first confirmed this result by applying our wave quantification method to a previously collected [27] EEG dataset (INPUT) composed of 20 participants, who fixated a 6.25-s-long white-noise sequence of random luminance values for about 360 trials (Fig 1A). For each electrode, we considered the cross-correlation averaged over all trials. We obtained the 2D map and computed the log ratio, as for the model, over 7 midline electrodes (Oz to Fz—see Fig 2A). Then, in order to estimate the proportion of reliable traveling waves, we computed the null distribution by shuffling the electrodes’ order 1,000 times for each subject before recomputing the log ratios. The difference between the real and the null distributions provides a statistical estimation of the proportion of reliable FW and BW wave events over and beyond what might be expected by chance, as captured by our surrogate distribution. In the INPUT dataset’s IRF, the comparison between the real and null distributions confirmed the presence of traveling waves (INPUT: Kolmogorov-Smirnov test, D = 0.507, *p* < 0.001, Fig 4B). Specifically, 56.6% of IRF data epochs showed evidence for FW waves over and beyond what could be expected by chance. In addition, the predominance of FW waves was further confirmed by testing the distribution of mean log ratios across participants against zero (Bayes factor [BF] = 32.4, error = 1.392% × 10^−4^, Fig 2D).

Once we confirmed the presence of FW traveling waves in the IRF measurements, we explored in the same dataset whether we could also demonstrate traveling waves in the raw EEG signals, as suggested by our model. Although several studies have reported traveling waves using intracortical recordings [13], there is so far little evidence from EEG studies [28,29]. Here, we applied a sliding window of 1 s (500-ms overlap) to the continuous EEG data and obtained approximately 4,000 epochs per subject. For each epoch, we computed the log ratio as for the IRF data and again compared the real and null distributions. Remarkably, the Kolmogorov-Smirnov test used to assess a difference between the two distributions revealed the presence of traveling waves in the EEG data (INPUT: D = 0.1217, *p* < 0.0001; Fig 4D). Particularly, 36.5% of the tested 1-s EEG epochs showed reliable evidence in favor of FW waves, against only 13.4% of BW waves. The bias in favor of the FW propagation was confirmed by a Bayesian *t* test between FW and BW waves (BF = 134.3, error = 1.055% × 10^−6^; see also S2A Fig). This result proves that direct EEG recordings can also reveal the presence of traveling waves, as suggested by the model’s simulations. Moreover, in line with the results using IRFs, the EEG data confirm that the bottom-up dynamics of perceptual processing are more strongly associated with FW than with BW traveling waves.

As previous studies showed that eye movement artifacts may modulate alpha-band activity [30–32], in a control analysis, we compared the log ratios of the 20 trials with the highest horizontal electrooculography (HEOG) activity with the 20 trials with the lowest activity. Similarly, we selected and compared 2 sets of 20 trials on the basis of the vertical electrooculography (VEOG) activity. The results (S7 Fig) showed no difference between the two subsets of trials, for either VEOG or HEOG activity, thus ruling out possible confounds due to eye movements (Bayesian paired-sample *t* test, HEOG: BF = 0.12; VEOG: BF = 0.27).

#### BW traveling waves in resting-state EEG data

Our model simulations revealed that predominantly FW waves during stimulus processing are replaced by BW waves in the absence of stimulus. With the purpose of assessing the presence of BW waves in human EEG signals, we thus turned to a second dataset (CLOSED EYES) composed of 48 participants, who underwent a 1-min recording with closed eyes. As for the previous dataset, we estimated the real and null log-ratio distributions after having computed the 2D maps for every 1-s time window (500-ms overlap), over the same line of 7 central electrodes (Oz to Fz). Eventually, we counted about 130 log-ratio values per subject. Notably, also in this dataset, the comparison between the two distributions unveiled the presence of traveling waves (CLOSED EYES: D = 0.2047, *p* < 0.0001; Fig 4D) revealing, as predicted by our simulations, a preponderance of BW over FW waves (respectively 41.1% and 8.8% of the tested 1-s EEG epochs), as confirmed by a Bayesian *t* test between FW and BW waves (BF = 74.2, error = 2.632% × 10^−8^; see also S2A Fig). All in all, this result corroborates the predictions of our model regarding (1) the possibility to reveal traveling waves from raw EEG signals and (2) the presence of BW waves in the absence of sensory stimulation.

Finally, we tested whether the waves occur preferably along the occipito-frontal axis or whether they can be observed in other orientations as well. We thus analyzed the presence of traveling waves along the left-to-right axis of the central electrodes (C5, C3, C1, Cz, C2, C4, C6). As shown in S9 Fig, the results demonstrate that the left-to-right axis produces significantly fewer waves (approximately 13% against 36% and 41%, respectively, in the INPUT and CLOSED EYES), equally likely in both directions (left to right or right to left), and independent of the stimulus’s presence.

#### Human/model comparisons

For the sake of comparison, we applied the same quantitative analyses to the modeling data as to the human EEG/IRF, obtaining qualitatively similar results. Notably, in the simulations, we were able to compute an IRF both with the input and with the top-down prior signals (for human experiments, we only have direct access to the visual input signals, but the internal priors, if any, remain unknown). In all simulations, significant traveling waves were observed both in IRF data (D = 0.9800, *p* < 0.0001; Fig 4A) and in EEG data (D = 0.7982, *p* < 0.0001; Fig 4C). Similar to the CLOSED EYES condition of the human EEG dataset, when only the top-down prior signal was present in the model, we observed exclusively BW waves: 79.3% of the tested 1-s EEG epochs showed evidence for BW waves, as did 100% of the IRF measurements (0% of EEG epochs or IRF measurements suggested FW waves). Conversely, when only the input was provided to the model, only FW waves were observed, in line with the results of the INPUT human EEG dataset: 76.8% of EEG epochs and 100% of IRF measurements revealed FW waves (with 0% of EEG epochs or IRF measurements supporting BW waves).

## Discussion

**Fig 5:**
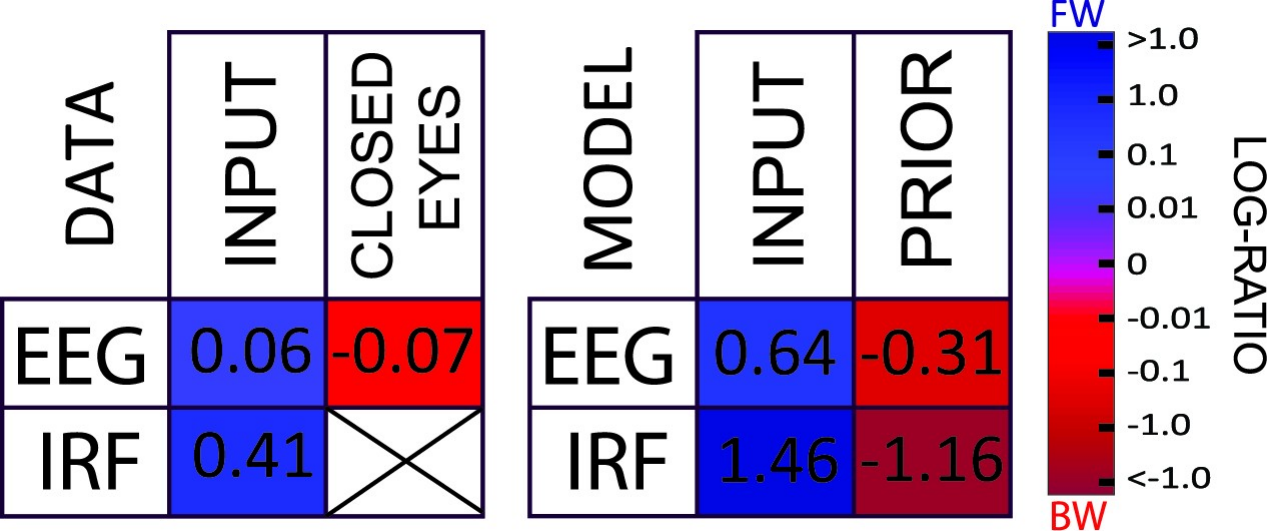
Summary of the results. The tables summarize the results of the data (left) and simulation (right) for each dataset/condition. In each situation, the average log ratio is presented numerically as well as color-coded. Red and blue squares indicate respectively a higher incidence of BW and FW waves. (As explained previously, it is not possible to compute an IRF in the CLOSED EYES condition.) All data are available at https://osf.io/nc4rg/?view_only=bb06fe996d1f49b285dc25464eec7ac7. BW, backward; EEG, electroencephalography; FW, forward; IRF, impulse response function.

We presented a novel hypothesis suggesting that the ubiquitous alpha rhythm could reflect, in part, the computations involved in predictive coding. Long-lasting (around 1 s) alpha-band oscillatory reverberation of visual inputs, compatible with experimental observations [9], could be reproduced in a simple model with only short-term dynamics—each neuron only integrates information over approximately 20 ms (neural time constant τ), and the delays for information transmission (ΔT) are also restricted to <20 ms. A systematic exploration of parameter space in both the 2-level and in the multilevel models revealed that IRF oscillations in the alpha (8–12 Hz) frequency range systematically arise when τ and ΔT lie in their “biologically plausible” range. Therefore, we conjecture that it may simply not be possible for a biological brain, in which communication delays are non-negligible, to implement predictive coding without also producing alpha-band reverberations (see, however, Perrinet and colleagues [33] for a model of active inference taking into account physiological delays in oculomotor control for predictable motion trajectories). Moreover, a major characteristic of alpha-band oscillations—i.e., their propagation through cortex as a traveling wave—could also be explained by a hierarchical multilevel version of our predictive coding model. The waves predominantly traveled FW during stimulus processing and BW in the absence of inputs. These simulation results were remarkably matched by our human EEG data analyses (Fig 5 provides a summary of the results) and are compatible with observations from other recent experimental studies [12,14,34].

### Generation of alpha (bidirectional interaction)

One of our simulations’ result refers to the generation of oscillations within a predictive coding framework enforced with temporal delays [35]. Specifically, our model posits that the bidirectional interaction between levels, influenced by communication delays, produces alpha-band rhythms. Electrophysiological studies have pointed at the origin of the alpha rhythms in the thalamic-cortical networks [36–38], particularly involving the activity of the thalamic reticular nucleus [39]. The results of our 2-level model (and the systematic exploration of its 2 free parameters τ and ΔT) may comply with this hypothesis by considering, respectively, the lower and higher level of the model as the thalamus and the primary visual cortex. However, other experimental evidence suggests that part of the alpha spectral power originates exclusively from cortical dynamics [12,39]: in particular, pyramidal neurons in infragranular and supragranular layers of V2 and V4 have been implicated as alpha pacemakers [37]. Similarly, other cortical regions (such as V1 or S1) revealed the presence of alpha current generators, involved in both feedforward and feedback processes [40–42]. The notion that alpha rhythms arise from the interaction of cortical levels is also in line with the results of our model, considering each of its levels as a different cortical region. Altogether, our model suggests predictive coding as a common computational framework involved in the generation of alpha oscillations, thus reconciling several apparently conflicting experimental findings on the origin of alpha.

### Types of traveling waves

Much work has been carried out regarding traveling waves, both in humans (mostly but not exclusively intracortical recordings, [13]) and in animals (e.g., turtles [43–44]; cats [45–46]). Specifically, 3 different types of dynamic spatial propagation of brain signals have been characterized in the literature and indiscriminately referred to as “traveling waves.” First, scalp event-related potentials (ERPs) propagating from posterior to anterior regions in response to stimulus onset have been interpreted as nonperiodic traveling waves [47–49]. Second, periodic sensory stimulations can produce oscillatory traveling waves that are confined to the same frequency as the entraining stimulus [50]: even though they behave rhythmically, these waves are similar in principle to the first type above, as their oscillatory nature is not necessarily intrinsic but is directly related to the frequency characteristics of the generating stimulus. Lastly, truly periodic traveling waves have their own intrinsic frequency (e.g., within the alpha-band range, [51]), unrelated to the frequency content of the stimulus (if any). In this study, we investigated this last type of traveling waves to understand the functional properties of intrinsically generated alpha-band oscillations.

### Generation of traveling waves

Ermentrout and Kleinfeld identified 3 different types of computational mechanisms that could lead to the generation of electrical traveling waves [51]. The first mechanism produces waves by virtue of a single neuronal oscillator, which spreads its rhythmic activity to neighboring and more distal regions with increasing time delays. In the brain, a potential candidate for such a role of pacemaker could be the above-mentioned cortical-thalamic circuit [37,52–54]. The second mechanism also suggests that the oscillatory wave originates as a result of a single oscillator, serially linked to other groups of neurons; however, whereas in the first model the wave’s motion was entirely due to varying communication delays from one oscillating region to all the others, in the second one the waves actually need to run through each region serially, with an increasing phase delay determined by the transmission delay between neighboring regions. Lastly, in the third mechanism, each region is modeled as an independent oscillator with its own frequency and phase. In agreement with the Kuramoto model [55], in this scenario the wave propagation follows the phase shifts between each region, which in turn depends on their relative frequencies, traveling from higher to lower frequencies. Remarkably, despite its numerous assumptions (i.e., weak coupling between regions, nearly identical oscillatory properties across regions), this last model [55] has been successfully applied in several computational brain studies [56,57]. Although our model does not exactly fit within any of these 3 categories, it does share some similarities with the last 2. On the one hand, the local interactions between neighboring regions with a constant communication delay relates to the second approach; on the other hand, the movement of the traveling wave arises naturally as in the last model from the communication between separately oscillating levels. Importantly though, in our model none of the units is an oscillator or pacemaker per se, but oscillations arise from bidirectional communication with temporal delays (see [58] for a similar approach to oscillations' generation). This property marks a decisive difference with all 3 previous models. Lastly, in our implementation, the wave’s direction does not depend on a gradient of time delays or a gradient of frequencies; instead, the same model can produce both FW or BW waves depending on the signals provided: FW waves originate from perceptual inputs, and BW waves originate from top-down activity. Regarding the anatomical origin of the traveling waves, it is possible to envision the first level of the model as a thalamic region (e.g., LGN) connected to the cortex or the entire hierarchy as reflecting successive cortical regions (e.g., V1, V2, etc.). In the brain, these regions would also interact with each other through indirect cortico-cortical connections, as well as via the thalamus (e.g., pulvinar); however, for simplicity, these secondary connection pathways were omitted in the model.

### Functional role of traveling waves

The traveling direction of the waves appears important in determining their functional role. In a recent study [12], intracortical recordings from epilepsy patients during quiet wakefulness revealed alpha-band traveling waves propagating “backward” from higher-order antero-superior cortex to lower-order occipital poles. Analyzing recordings simultaneously from cortex and the pulvinar, Halgren and colleagues concluded that alpha-band traveling waves (1) originate in the cortex and (2) reflect feedback processing between cortical regions. However, another recent human intracranial study from Zhang and colleagues [14] reported theta-alpha traveling waves (2–15 Hz) that could propagate “forward,” from posterior to anterior brain regions, during the cue period of a visual memory task. Although these two studies appear contradictory in terms of the propagation direction, our model can easily reconcile these findings by positing that alpha-band traveling waves emerge as a result of level-by-level interactions in a hierarchical system, and their direction is related to their functional role: FW to convey input signals (and the prediction residuals) during sensory processing and task engagement and BW to convey top-down priors and expectation signals, most visible during rest and in the absence of inputs. One could ask what the relationship is between alpha-band traveling waves and other putative functions usually related to alpha rhythms, such as attention. Although the classic view suggests that alpha rhythms tend to decrease under the effect of attention [2,59,60], a previous study demonstrated that alpha-band perceptual echoes are positively related with attention allocation. Consequently, it is possible to speculate that FW waves (which are associated with stimulus processing) would positively correlate with increases in attention allocation. Conversely, the BW waves we observed in the closed-eyes condition could be related with the putative top-down processing related to alpha-band activity in sensory tasks [61], temporal prediction [62], and visual attention [63].

### Speed of traveling waves

The speed of cortical traveling waves appears to depend on several factors, as reflected by the large range of values reported in the literature (from 0.1 to 10 m/s). One important distinction regards whether the traveling waves are recorded at a macroscopic (i.e., whole-brain) or mesoscopic level (i.e., within single regions in the cortex) [13]. In the first case, magnetoencephalography (MEG), EEG, or electrocorticography (ECoG) recordings allow determination of their propagation, which supposedly occurs through myelinated axons of white matter fibers: consequently, their speed spans between 1 and 10 m/s [13,29]. Conversely, techniques with higher spatial resolution, such as voltage-sensitive dye imaging [64] or multielectrode arrays [65], grant access to local, slower traveling waves whose speed ranges from 0.1 to 0.8 m/s [14,55], in agreement with the axonal conduction speed of unmyelinated long-range and horizontal fibers within the cortex [66]. In addition, another important factor potentially affecting the measured traveling waves speed is cortical source mixing, which is especially pronounced when recording from the scalp (i.e., MEG, EEG): as every recording channel picks up not only local but also more distal cortical signals, we should expect an apparently faster speed at the scalp level than the true underlying speed of the traveling wave over the cortex. This could explain why the waves seem to traverse the 7 levels of our model much more slowly than they go through our 7 scalp-level electrodes in the human EEG recordings (compare Fig 2A to Fig 3B). In support of this view, we simulated EEG source mixing in our model to assess the corresponding changes in wave speed and then compared the results with real EEG data (see S3 Fig). By assuming a level-to-level distance of 2 cm, roughly equivalent to the distance between neighboring cortical regions, our model produces traveling waves whose speed falls in the mesoscopic range (approximately 0.6 m/s, see S3 Fig), comparable with experimental cortical recordings [14,55]. However, after remapping the model deep levels to superficial electrodes by means of a weighted average (thus simulating cortical source mixing, S3B Fig), the speed of the traveling waves increased, now falling within the range of macroscopic waves (approximately 2.2 m/s). Importantly, this linear averaging, despite increasing the apparent speed, did not significantly affect the waves’ direction or their log ratios. Furthermore, the speed of the scalp-level traveling waves as simulated by our model was now comparable with the one observed in our experimental human EEG dataset (approximately 2.2 m/s, see S3D Fig).

### Relation to other predictive coding models

Because of its streamlined architecture, our predictive coding model produces a single oscillation whose frequency depends on the chosen parameters: typically in the alpha band for biologically plausible values (Fig 1D). The same alpha oscillation thus carries top-down predictions down the hierarchy and bottom-up prediction residuals up the hierarchy, resulting in 2 alpha traveling waves moving in opposite directions. As explained above, this is compatible with recent experimental reports of both feedforward and BW alpha traveling waves in human intracranial studies [12,14]. However, there is also a growing number of studies reporting that faster gamma oscillations (about 30–80 Hz) are specifically involved in feedforward signal transmission, whereas alpha- and beta-band (13–30 Hz) rhythms convey top-down information [41,67–72]. These dynamics have been appropriately captured by a more detailed predictive coding model, the “canonical cortical microcircuit” model [73–74], which includes different types of excitatory and inhibitory neurons, as well as detailed laminar circuitry in each brain region. An important next step would thus be to explore the existence, frequency, and direction of traveling waves in a hierarchical version of the canonical microcircuit model. One can speculate that such a model could account both for the bidirectional nature of alpha traveling waves across the hierarchy (as observed in our model and in the above-cited human intracranial studies) and for the prevalence of gamma-band signals in the feedforward communication between sending and receiving layers of 2 consecutive brain regions (as measured, e.g., using Dynamic Causal Modelling in ECoG data in monkeys, see [74]). Finally, future versions of our model could also expand the number of simulated neurons in each level, together with a retinotopic organization and spatially selective receptive fields (as in [1]), to process spatially as well as temporally structured inputs. This should provide a more complete understanding of predictive coding and oscillatory traveling waves in relation to essential visual functions such as object recognition or categorization.

## Materials and methods

### Ethics statement

All participants gave written informed consent before starting the experiment, in accordance with the Declaration of Helsinki. This study was carried out in accordance with the guidelines for research at the “Centre de Recherche Cerveau et Cognition,” and the protocol was approved by the committee “Comité de protection des Personnes Sud Méditerranée 1” (ethics approval number N° 2016-A01937-44).

### Model and simulations

Predictive coding [1] postulates a hierarchical architecture in which higher levels predict the activity of lower levels, and the residuals (i.e., the difference between the prediction and the actual activity) are carried over to update the next prediction. In our model, illustrated in Figs 1B and 3A, the residual is defined as:

$$x_{L}\left(t\right)=y_{L-1}\left(t\right)-y_{L}\left(t-\Delta T\right)$$ (1)

where L indexes the levels, and ΔT represents the temporal communication delay between them. For simplicity, the delay is assumed here to be symmetric in both FW and BW directions, but it can easily be shown that the oscillatory behavior chiefly depends on the sum of FW and BW delays (e.g., comparable oscillatory dynamics would be found for symmetric delays with ΔT = 12 ms, or for asymmetric delays with ΔT_forward_ = 16 ms and ΔT_backward_ = 8 ms). For the consistency of notations, we pose y_L − 1_(t) = INPUT(t) when L = 1. The prediction y_L_, as shown in Eq 2, is updated based on the bottom-up residual x_L_ (with a delay ΔT) and on the difference between its prediction and the prediction from the next higher level, which can be considered as a top-down prior:

$$\frac{{\mathrm{dy}}_{L}}{\mathrm{dt}}=\frac{1}{\tau }\times x_{L}\left(t-\Delta T\right)+\frac{1}{\tau_{D}}\times \left(y_{L+1}\left(t-\Delta T\right)-y_{L}\left(t\right)\right)$$ (2)

At the last level, the prior y_L+1_ can serve to represent a generative endogenous process, arising from higher-level brain regions that are not part of the model; in our simulations, this top-level prior could be imposed as a time-varying signal or set to 0 (see Fig 3A). In order to facilitate measurements of cross-correlations (IRF) with this prior and comparison with the stimulus-induced IRF, when different than 0 we set the time-varying top-level prior to a white-noise signal with statistics similar to (but independent from) those of the input signals.

Besides ΔT, 2 other parameters play a crucial role in the model: τ and τ_D_, which describe the temporal dynamics (time constants) of neuronal integration and decay, weighting respectively the residual computed from the lower level and the prediction from the higher level. Note that when the prior y_L+1_ is set to 0, the second term in Eq 2 acts as a decay term, which would ensure that the prediction y_L_(t) returns to zero in the absence of inputs. For this reason, and to take into account the fact that higher-level brain signals typically vary slower than low-level input signals [22,24–26], we set the time constant τ_D_ to a fixed value of 200 ms. In all simulations, Eqs 1 and 2 were solved numerically with a 1-ms time step.

The results of the parameter search in the 2-level (Fig 1D) model were obtained presenting, for each pair of τ and ΔT, 200 different white-noise luminance sequences of 3 s. The model’s IRF was computed by cross-correlating each luminance sequence with level 2’s output y(t) and averaging the results over the 200 trials. Regarding the results of the multilevel model (Fig 4A and 4C), we investigated 2 possible scenarios, in which either an input or a prior signal was presented to the model for 6 s on each of 200 simulated trials. Consequently, we computed the IRF by cross-correlating each level’s output y_L_(t) with one of the two signals. We then defined 2D maps (Fig 2A and 2B) stacking up the temporal signals from all 7 model levels. We thus obtained two 2D maps, 1 for each scenario, (i.e., IRF with input or IRF with prior). Moreover, we computed 2 additional 2D maps considering the raw (simulated) EEG signals (no cross-correlation) in place of the IRF, separately for each scenario (in this case, the temporal signals were obtained with a 1-s sliding window with 500-ms overlap, yielding 11 distinct analysis windows for each trial). For each of these maps, we then computed a log ratio that quantifies the presence and the direction of the waves (see below). Log-ratio distributions were obtained by pooling together the results over the 200 simulated trials. We followed the same procedure for the null distribution, obtained by randomly shuffling the order of the levels before computing the log ratios. During the parameter exploration in the multilevel model (Fig 3C and 3D), we performed the same analysis as described above, computing log ratios, their frequencies, and spectral power for both FW and BW waves (i.e., for opposite quadrants of the 2D FFT) in both IRF and EEG maps. In two different analyses, we explored the traveling waves’ dynamics, first by varying τ and ΔT pairs in the range of 1–30 ms and then by varying τ_D_ over the range of 20–200 ms. The code of the model and of all simulations is available at https://github.com/artipago/echoPred.

### Analytical solution of the (simplified) model

An analytical solution for our system of delayed differential Eqs 1 and 2 exists [75] for a 2-level model under 2 simplifying assumptions: (1) we neglect the second term of Eq 2, given that τ_D >>_ τ; (2) the integral over time of the input signal amounts to 0 (e.g., white noise centered on 0). This leads to the following equation, obtained by merging the simplified Eqs 1 and 2:

$$\frac{dy}{dt}=-\frac{1}{\tau }y(t-2\Delta T)$$ (3)

If we consider as a general solution the exponential function in Eq 4, we can compute each side (respectively Eq 5 and Eq 6) of Eq 3 as

$${y(t)=e}^{\alpha t}$$ (4)

$$\frac{dy}{dt}={\alpha e}^{\alpha \;t}$$ (5)

$${y(t-2\Delta )=e}^{\alpha \;t}e^{-2\alpha \Delta T}$$ (6)

By replacing Eq 5 and Eq 6 in Eq 3, we obtain Eq 7, simplified in Eq 8:

$${\alpha e}^{\alpha \;t}=-\frac{1}{\tau }e^{\alpha \;t}e^{-\alpha \;2\Delta T}$$ (7)

$$-\alpha \tau =e^{-\alpha \;2\Delta T}$$ (8)

A “pure” oscillatory solution would correspond to a situation in which α is an imaginary complex number, that is, α = 0 + iω (where ω is the oscillatory frequency). Given Eq 8, this leads to:

$$-i\;\omega \;\tau =e^{-i\;\omega \;2\Delta T}$$ (9)

Eventually, applying Euler’s formula, we derive 2 equations by separately considering the real Eq 10 and the imaginary Eq 14 parts. Since the real part is equal to 0, we obtain

cos⁡(2ωΔT)=0 (10)

$$2\omega \Delta T=\frac{\pi }{2}+k\pi \;for\;k∊Z$$ (11)

$$\omega =\frac{2\;\pi }{8\;\Delta T}+\frac{4\;k\;\pi }{8\;\Delta T}\;for\;k∊Z$$ (12)

whose first oscillatory solution (with lowest frequency) is

$$\omega =\frac{2\;\pi }{8\;\Delta T}$$ (13)

In plain English: if the model solution is oscillatory, it will most likely oscillate with a period around 8ΔT.

Regarding the imaginary part, we can write

ωτ=sin⁡(2ωΔT) (14)

which, given Eq 10, must equal 1 or −1. As both ω and τ are positive, we conclude that

$$\omega =\frac{1}{\tau }$$ (15)

Combining Eq 13 with Eq 15 provides Eq 16 and 17:

$$\tau =\frac{8\;\Delta T}{2\;\pi }$$ (16)

τ≈1.2732ΔT (17)

In conclusion, oscillatory solutions exist in a region of parameter space (ΔT, τ) slightly above the diagonal (τ = ΔT). The period of the oscillation is 8ΔT, which will lie in the alpha band (8–13 Hz) whenever ΔT is between 10 and 15 ms. In that case, Eq 17 suggests that τ should be around 13–20 ms. Therefore, the equations theoretically confirm the results of the numerical parameter search (Fig 1D), defining the region of parameter space where solutions oscillate in the alpha band.

## Experimental datasets

### Participants

The INPUT dataset was composed of EEG recordings from 20 volunteers (aged 23–39 y with a mean age of 28 y, 10 women, 5 left-handed), the CLOSED EYES dataset from 48 volunteers (aged 20–43 y with a mean age of 27.8 y, 25 women, 7 left-handed). All subjects reported normal or corrected-to-normal vision and no history of epileptic seizures or photosensitivity.

### Stimuli and protocols

Results from the INPUT dataset have been published elsewhere [27,76], whereas the second, the CLOSED EYES dataset, presents a mixture of published and unpublished data. Regarding the INPUT dataset, the experiment was composed of 2 sessions of 8 experimental blocks of 48 trials each, having a total duration of about 1 h. Participants viewed 6.25-s-long random (white-noise) luminance sequences presented on a cathode ray monitor, positioned roughly 57 cm from the subject, with a refresh rate of 160 Hz and a resolution of 640 × 480 pixels. Each sequence had a flat power spectrum up to 80 Hz and was designed using MATLAB custom scripts and displayed using the Psychophysics Toolbox [77]. The stimuli were presented on a black background in a peripheral disk with a diameter of 7°, whose center was at 7° of eccentricity above the fixation point. Concerning the CLOSED EYES dataset, participants were asked to close their eyes for 1 min while recording their spontaneous brain activity (this is done routinely in our lab to measure each participant’s individual alpha frequency). After the recording ended, participants performed other tasks unreported in this manuscript.

### EEG recording and preprocessing

In both datasets, brain activity was recorded using a 64-channel active BioSemi EEG system (1,024 Hz digitizing rate, 3 additional ocular electrodes). The following preprocessing steps were applied to all subjects of the INPUT dataset using the EEGlab toolbox [78] in MATLAB. Once the noisy channels had been rejected and interpolated (when necessary), the data were offline down-sampled to 160 Hz to match the presentation rate of luminance stimuli and thus facilitate the cross-correlation of the two signals. A notch filter (47–53 Hz) was then applied to remove power line artifacts. We applied an average-referencing and removed slow drifts by applying a high-pass filter (>1 Hz). In the first dataset (i.e., INPUT), data epochs were created around each white-noise sequence (from −0.25 to 6.5 s) and the baseline activity was subtracted (i.e., mean activity from −0.25 s to 0 before trial onset). Finally, the data were screened manually for eye movements, blinks, and muscular artifacts, and whole epochs were rejected as needed: on average, 20/384 trials were rejected per subject. For the second dataset (CLOSED EYES), preprocessing was limited to noisy channels rejection, and a similar filtering process as in the previous dataset was performed: after a notch filter (47–53 Hz) was applied, a high-pass filter (>1 Hz) removed slow drifts. All data are publicly available at https://osf.io/nc4rg/?view_only=bb06fe996d1f49b285dc25464eec7ac7.

### Log ratio

We computed log ratios of each 2D map (Fig 2A and 2B) in order to assess and quantify the presence of traveling waves in both EEG signals and simulations. To create 2D maps of the human EEG data, we considered either the raw EEG signals from 7 midline electrodes (posterior to frontal: Oz, POz, Pz, CPz, Cz, FCz, Fz—importantly, the electrodes’ choice does not reflect any assumption about the exact localization of the waves, thus not implying a dorsal rather than ventral propagation; for details, see [10]) or their cross-correlation with the input sequence (i.e., IRFs). Regarding the simulations, we utilized either the prediction signals from each level of the model or their cross-correlation (IRF) with either the input or the prior signals. Each 2D map for the raw EEG (or its model equivalent) was computed with a sliding window of 1 s and an overlap of 500 ms (x-axis). The 2D maps for IRFs were obtained directly by stacking the 7 IRF time courses (from 7 EEG electrodes or from 7 model levels), as the support window of the IRF was only 1 s long. The maximum value of the upper-left quadrant of the 2D FFT was extracted to represent the amount of FW signal propagation across levels/electrodes, whereas the maximum value of the lower-left quadrant revealed the number of BW waves. Finally, we computed the log ratios by dividing the FW maximum value by the BW maximum value and taking the log of the result: positive and negative ratios revealed, respectively, FW and BW waves. A log ratio equal to zero could indicate either an equal amount of FW and BW waves or a “standing wave” that does not travel in either direction, i.e., a globally synchronized rhythm (this would happen when the maximum spectral value in each quadrant corresponds to the same spatial frequency of zero, i.e., the horizontal midline of the 2D FFT, which we had included in both 2D FFT quadrants; note that we also performed this analysis with max spectral values, ignoring the zero-spatial-frequency axis: results were qualitatively similar, indicating that stationary rhythms are not prominent in our dataset).

## Supporting information

S1 Fig Overview of 2D maps from all the simulations and experimental data. (A) The picture reports all the possible simulation scenarios. The left column shows the 2D maps of the IRF, whereas the right column shows the 2D maps of the EEG (i.e., the prediction signals from each model level). Each row shows the results of a different simulation, with either the input signal (first row), the top-down prior signal (last row), or both together (middle panels; although there is only 1 EEG signal in this situation, it can be cross-correlated with either the input or the prior signals, hence the 2 panels in the left column). (B) The first row shows IRF (left) and EEG (right) 2D maps from 1 representative participant in the INPUT dataset. The second row shows another representative participant in the CLOSED EYES dataset; only the EEG map is displayed, since we do not have access to either external input signals or internal top-down priors to cross-correlate with the EEG and derive an IRF. EEG, electroencephalography; IRF, impulse response function.
(TIF)

S2 Fig Direction of traveling waves in human EEG data. The first 2 panels show the proportion of BW (in red) and FW (in blue) traveling waves, respectively, in the INPUT and CLOSED EYES dataset for each participant, as computed in Fig 4D. Each value represents the percentage of waves that occur above chance level, when compared to the null distribution (see Fig 4 and Materials and methods for details). In the INPUT dataset, most participants have a larger number of FW waves than of BW, whereas the CLOSED EYES dataset reveals an opposite trend. The mean ± SE log ratios of both datasets result corroborated this result, as shown in the right panel. See S2 Data. BW, backward; EEG, electroencephalography; FW, forward.
(TIF)

S3 Fig Speed of traveling waves. (A) Instead of assuming that distinct model levels correspond to distinct scalp-level electrodes, each level of the model can be interpreted as a cortical brain region and, consequently, each prediction sequence as a cortical recording. Under these assumptions, the speed of the traveling wave, computed assuming a distance of roughly 2 cm between neighboring cortical regions, is about 0.6 m/s (model EEG in the picture, but similar results are obtained with IRF data). (B) Applying a moving linear weighted average for every level at each time point (the red box highlights an example) determines a transformation in which each new level (i.e., electrode) is influenced by neighboring levels, in the same way as superficial EEG electrodes are presumably affected by multiple deep cortical sources. In this transformation, the contribution of each level is weighted based on its distance to the electrode. The contribution of the kth level for the kth electrode is equal to 1, and it decreases progressively with distance to 0.8, 0.6, and 0.4 (respectively for levels k ± 1, k ± 2, and k ± 3). The contribution of levels further away was set to 0. (C) The transformed map reveals a traveling wave whose speed is faster and more compatible with EEG recordings. Importantly, the wave direction and the log ratios are not significantly influenced by such transformation. (D) The traveling-wave speed of the model before (blue and violet) and after (green and cyan) the transformation, compared to the speed of all the significant waves in our experimental datasets (i.e., epochs whose log-ratio was above chance level, as estimated by the surrogate distributions). We estimated an average electrode-to-electrode distance of about 4 cm for the real data and the superficial model simulations and a level-to-level distance of about 2 cm for the deep model. See S3 Data. EEG, electroencephalography; IRF, impulse response function.
(TIF)

S4 Fig Multiregion model—Parameter search. (A) Systematic exploration of parameters ΔT and τ when the model was provided only with the INPUT (first row), only the PRIOR (second row), or both signals (lower part of the figure). In every simulation, τ_D_ was kept at 200 ms. Absolute log-ratio values below 0.2 were set to 0. In the left column, log ratios were computed on the 2D maps obtained by cross-correlating the input (or prior) with the prediction of each region. When only the INPUT was provided, log ratios are mostly positive (in yellow), whereas when only the PRIOR was fed to the model, we observed mostly negative values (in blue). When both inputs are jointly provided, the dynamics induced by the inputs appear to dominate, which is mathematically expected given τ_D_ >> τ; consequently, log ratios are mostly positive (bottom panels; although there is only 1 EEG signal in this situation, it can be cross-correlated with either the input or the prior signals, hence the 2 panels in the left column). In the right column, the same log-ratio analysis is directly applied to (simulated) EEG signals (1-s epochs), without performing a cross-correlation with inputs. (B) Same as (A) but showing the frequency of the 2D FFT spectral peak in place of log ratios. As in the 2-level model, the range 10 < ΔT < 15 and 15 < τ < 20 gives rise to oscillations in the alpha range. The code of the model and all simulations is available at https://github.com/artipago/echoPred. FFT, fast-Fourier transform.
(TIF)

S5 Fig Multilevel model—Parameter search over τ_D_. (A) The first row shows how the log ratio varies according to τ_D_ (when ΔT and τ are kept constant to 12 ms and 20 ms, respectively). The last two rows show how the maximum power value varies as a function of τ_D_. The first column shows the results when the model was fed only with the INPUT, in the second one only with the PRIOR, and both INPUT and PRIOR in the last one. Each value is averaged over 20 repetitions. (B) As in (A) but showing the peak spectral frequency of the traveling waves as a function of τ_D_. As expected, when τ_D_ is larger than τ, the oscillations falls in the alpha range. Globally, there are no qualitative changes in the model behavior for τ_D_ > 50 ms or so. The code of the model and all simulations is available at https://github.com/artipago/echoPred.
(TIF)

S6 Fig Temporal time constant in the simulated EEG. At each model’s hierarchical level, we computed the prediction’s autocorrelation. We then subsampled them (B) and fit each level with a decreasing exponential function. The temporal constants of the fits (i.e., θ) are shown in (C), revealing a steady increase as a function of the hierarchical level, in agreement with experimental observations [22]. Panel D displays the fitted curves. The color code in (B-D) spans from red to green colors according to the hierarchical level (red: first level; green: last level). See S4 Data. EEG, electroencephalography.
(TIF)

S7 Fig Control analysis: Influence of eye movements on traveling waves. We compared the log ratio between trials with highest versus lowest ocular activity, separately for the HEOG (in red) and VEOG (in blue). The colored lines represent individual participants, with the average ± SE of the mean in black. For both the VEOG and the HEOG, there is no systematic difference in the log ratio, as confirmed by a Bayesian paired-sample *t* test (both BF < 0.3). The log ratios are overall positive, indicating forward-traveling waves, independent of ocular activity. The HEOG was computed by subtracting the activity in 2 external electrodes set over each participant’s temples. The VEOG was computed by subtracting an external electrode located under the left eye with the signal recorded at the frontal electrode AFz. See S5 Data. BF, Bayes factor; HEOG, horizontal electrooculography; VEOG, vertical electrooculography.
(TIF)

S8 Fig Control analyses in the model. Simulation results when the predictions of every layer are passed through a nonlinear sigmoid function. Similarly to Fig 4 of the main manuscript, the first row shows the real (solid line) and shuffled (dashed line) distributions of log ratios computed over (1) the IRF (2 panels to the left) obtained by cross-correlating predictions with either the sensory inputs (first column) or with the top-down prior signals (second column) and (2) the prediction of the models (considered as a proxy for the EEG signal). The second row shows the (positive values of the) difference between the real and surrogate distributions, representing the traveling-wave events occurring more often than predicted by the null hypothesis. The proportion of significant backward and forward waves are shown in red and blue, respectively. The results of these nonlinear simulations are in line with the ones obtained with the linear system. All data are available at https://osf.io/nc4rg/?view_only=bb06fe996d1f49b285dc25464eec7ac7. EEG, electroencephalography; IRF, impulse response function.
(TIF)

S9 Fig Control analyses in human data. Results for the IRF and EEG from the human EEG datasets (both INPUT and CLOSED EYES) similar to Fig 4B and 4D of the main manuscript, but considering electrodes C5, C3, C1, Cz, C2, C4, C6 (instead of Oz, POz, Pz, PCz, Cz, FCz, Fz). The purpose of this control analysis was to test whether we could detect left-to-right or right-to-left traveling waves. Although the distributions of log-ratio values depart somewhat from the corresponding surrogate distributions (because of the spatial consistency of brain signals in the real data, but not in the surrogate data), there is no preferred direction either for the IRF (rightmost column) or for the raw EEG data (2 leftmost columns). All data are available at https://osf.io/nc4rg/?view_only=bb06fe996d1f49b285dc25464eec7ac7. EEG, electroencephalography; IRF, impulse response function.
(TIF)

S1 Data Excel file containing the numerical data values for Fig 2C (both forward and backward power values for each participant) and 2D (log-ratio values). (XLSX)

S2 Data Excel file containing the numerical data values for S2 Fig (forward, backward, and log ratio for both INPUT and CLOSED EYES datasets). (XLSX)

S3 Data Excel file containing the numerical data values for S3D Fig (average values and standard errors of the mean). (XLSX)

S4 Data Excel file containing the numerical data values of the time constant for each level in S6C Fig. (XLSX)

S5 Data Excel file containing the numerical data values in S7 Fig of both horizontal and vertical eye movements in low- and high-activity trials. (XLSX)

## Abbreviations

BF: Bayes factor
BW: backward
ECoG: electrocorticography
EEG: electroencephalography
ERP: event-related potential
FFT: fast-Fourier transform
FW: forward
HEOG: horizontal electrooculography
IRF: impulse response function
LGN: lateral geniculate nucleus
MEG: magnetoencephalography
VEOG: vertical electrooculography
