# Supplementary figures and images for: Alpha oscillations and traveling waves: Signatures of predictive coding?
Source: PLoS Biol. 2019 Oct 3;17(10):e3000487. doi: 10.1371/journal.pbio.3000487 (PMC6776260; doi:10.1371/journal.pbio.3000487)

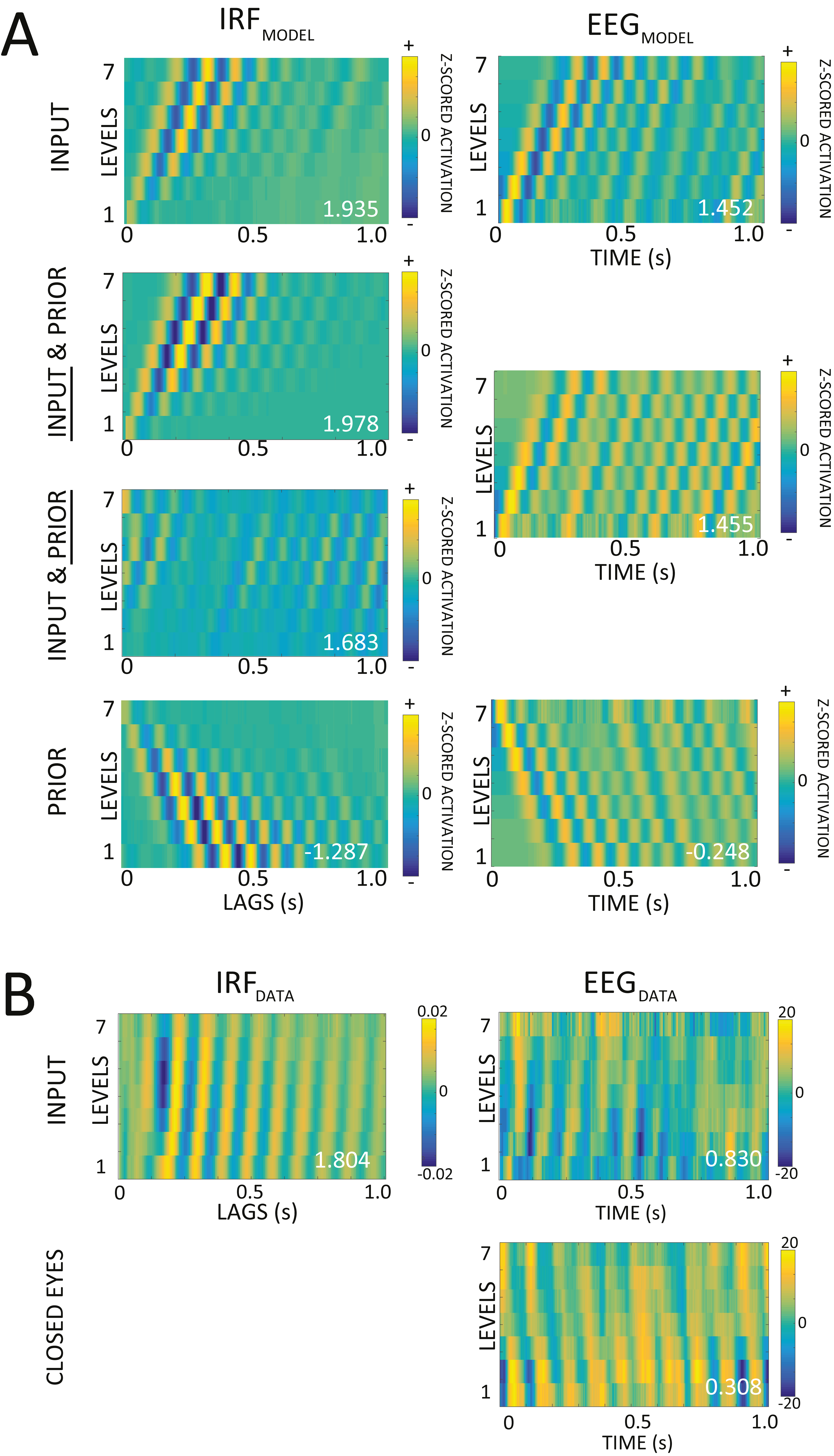

Supplement: S1 Fig — (A) The picture reports all the possible simulation scenarios. The left column shows the 2D maps of the IRF, whereas the right column shows the 2D maps of the EEG (i.e., the prediction signals from each model level). Each row shows the results of a different simulation, with either the input signal (first row), the top-down prior signal (last row), or both together (middle panels; although there is only 1 EEG signal in this situation, it can be cross-correlated with either the input or the prior signals, hence the 2 panels in the left column). (B) The first row shows IRF (left) and EEG (right) 2D maps from 1 representative participant in the INPUT dataset. The second row shows another representative participant in the CLOSED EYES dataset; only the EEG map is displayed, since we do not have access to either external input signals or internal top-down priors to cross-correlate with the EEG and derive an IRF. EEG, electroencephalography; IRF, impulse response function. (TIF) [file pbio.3000487.s001.tif]

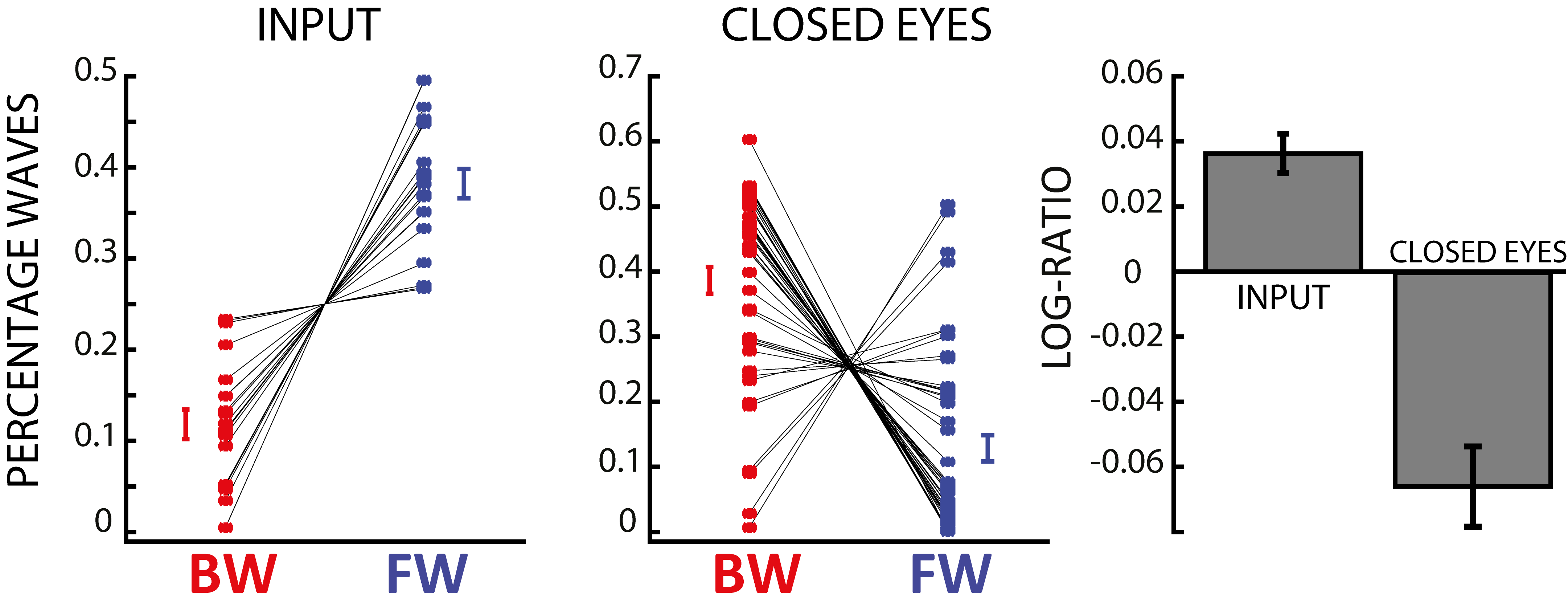

Supplement: S2 Fig — The first 2 panels show the proportion of BW (in red) and FW (in blue) traveling waves, respectively, in the INPUT and CLOSED EYES dataset for each participant, as computed in Fig 4D. Each value represents the percentage of waves that occur above chance level, when compared to the null distribution (see Fig 4 and Materials and methods for details). In the INPUT dataset, most participants have a larger number of FW waves than of BW, whereas the CLOSED EYES dataset reveals an opposite trend. The mean ± SE log ratios of both datasets result corroborated this result, as shown in the right panel. See S2 Data. BW, backward; EEG, electroencephalography; FW, forward. (TIF) [file pbio.3000487.s002.tif]

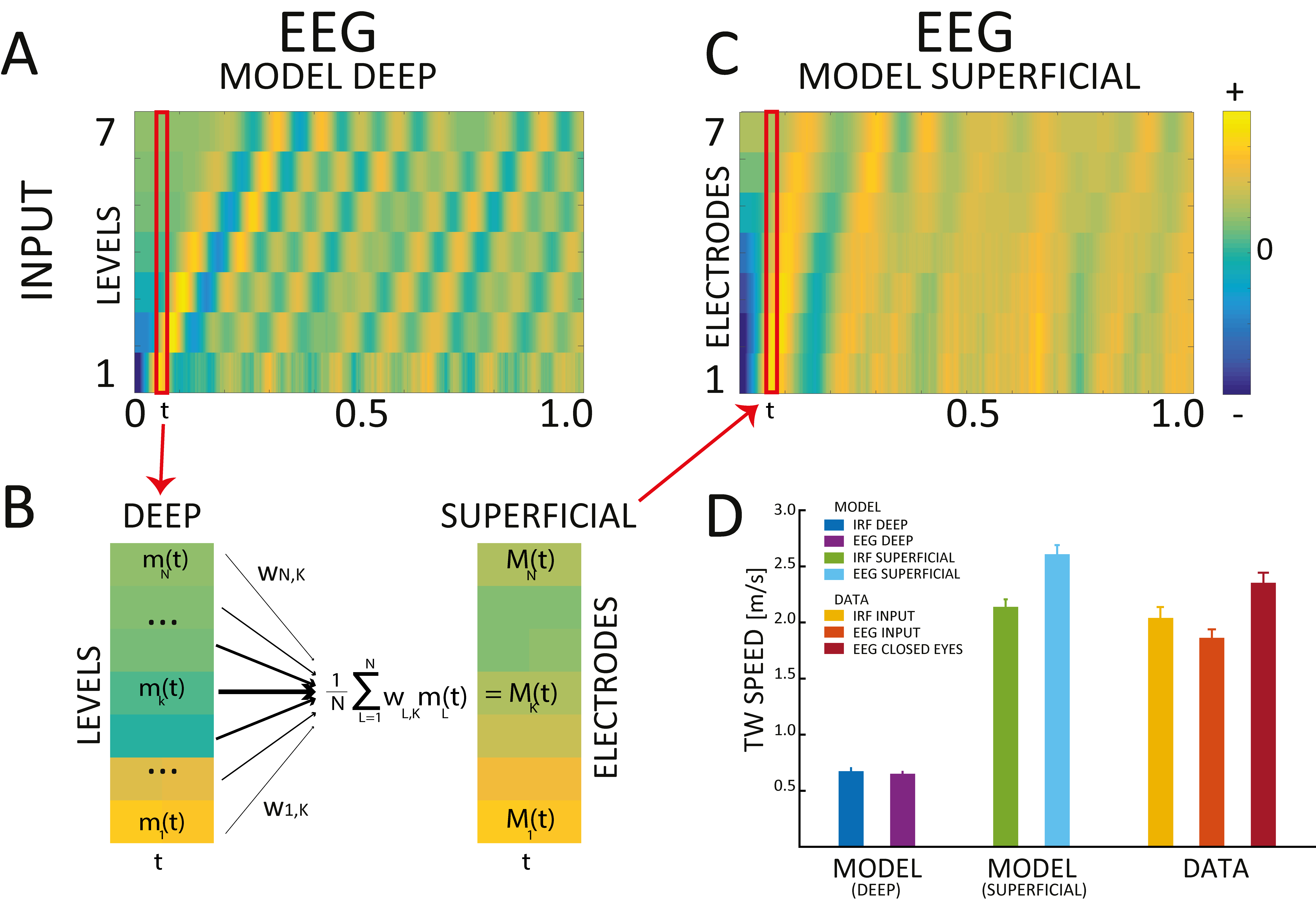

Supplement: S3 Fig — (A) Instead of assuming that distinct model levels correspond to distinct scalp-level electrodes, each level of the model can be interpreted as a cortical brain region and, consequently, each prediction sequence as a cortical recording. Under these assumptions, the speed of the traveling wave, computed assuming a distance of roughly 2 cm between neighboring cortical regions, is about 0.6 m/s (model EEG in the picture, but similar results are obtained with IRF data). (B) Applying a moving linear weighted average for every level at each time point (the red box highlights an example) determines a transformation in which each new level (i.e., electrode) is influenced by neighboring levels, in the same way as superficial EEG electrodes are presumably affected by multiple deep cortical sources. In this transformation, the contribution of each level is weighted based on its distance to the electrode. The contribution of the kth level for the kth electrode is equal to 1, and it decreases progressively with distance to 0.8, 0.6, and 0.4 (respectively for levels k ± 1, k ± 2, and k ± 3). The contribution of levels further away was set to 0. (C) The transformed map reveals a traveling wave whose speed is faster and more compatible with EEG recordings. Importantly, the wave direction and the log ratios are not significantly influenced by such transformation. (D) The traveling-wave speed of the model before (blue and violet) and after (green and cyan) the transformation, compared to the speed of all the significant waves in our experimental datasets (i.e., epochs whose log-ratio was above chance level, as estimated by the surrogate distributions). We estimated an average electrode-to-electrode distance of about 4 cm for the real data and the superficial model simulations and a level-to-level distance of about 2 cm for the deep model. See S3 Data. EEG, electroencephalography; IRF, impulse response function. (TIF) [file pbio.3000487.s003.tif]

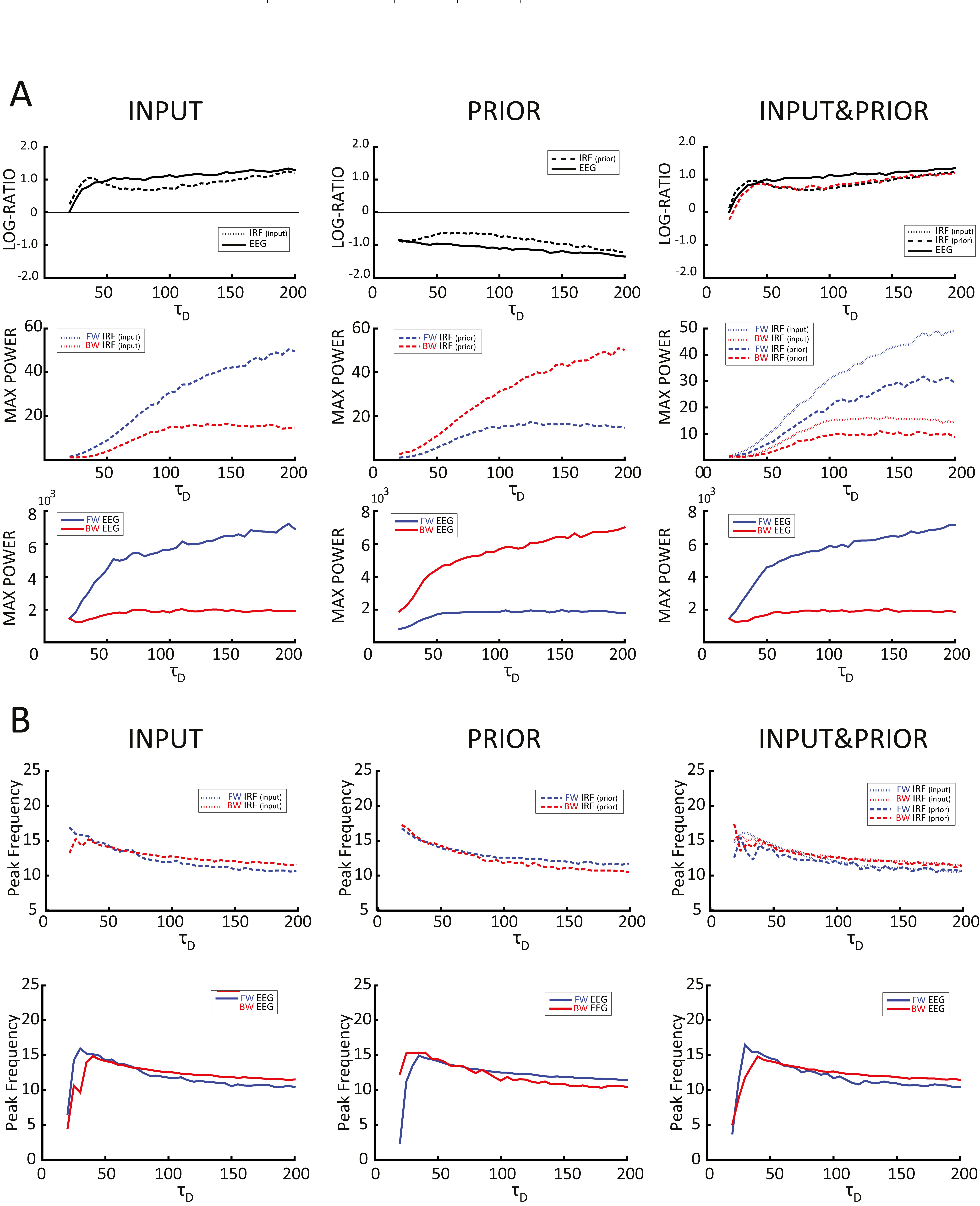

Supplement: S5 Fig — (A) The first row shows how the log ratio varies according to τD (when ΔT and τ are kept constant to 12 ms and 20 ms, respectively). The last two rows show how the maximum power value varies as a function of τD. The first column shows the results when the model was fed only with the INPUT, in the second one only with the PRIOR, and both INPUT and PRIOR in the last one. Each value is averaged over 20 repetitions. (B) As in (A) but showing the peak spectral frequency of the traveling waves as a function of τD. As expected, when τD is larger than τ, the oscillations falls in the alpha range. Globally, there are no qualitative changes in the model behavior for τD > 50 ms or so. The code of the model and all simulations is available at https://github.com/artipago/echoPred. (TIF) [file pbio.3000487.s005.tif]

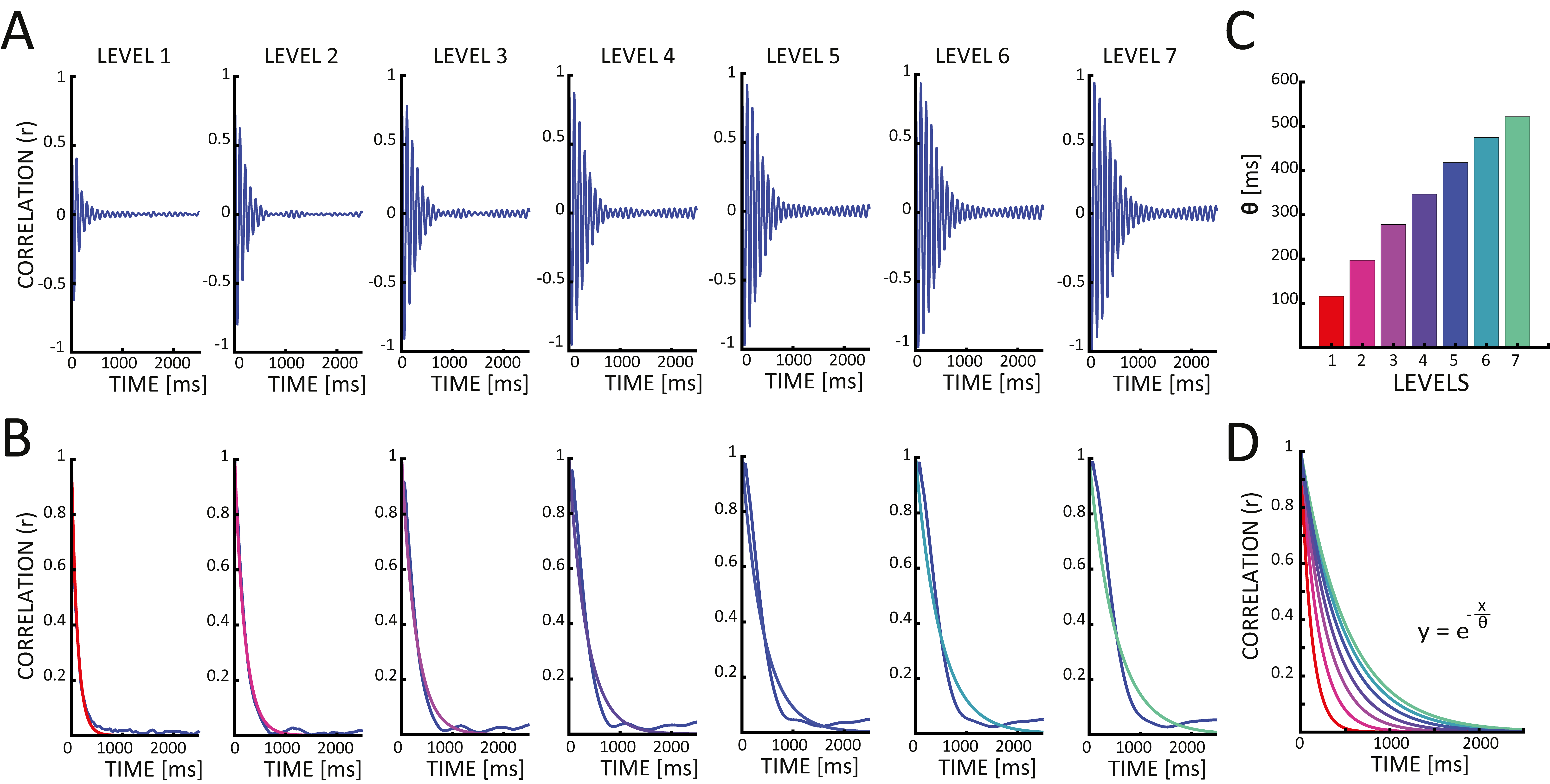

Supplement: S6 Fig — At each model’s hierarchical level, we computed the prediction’s autocorrelation. We then subsampled them (B) and fit each level with a decreasing exponential function. The temporal constants of the fits (i.e., θ) are shown in (C), revealing a steady increase as a function of the hierarchical level, in agreement with experimental observations [22]. Panel D displays the fitted curves. The color code in (B-D) spans from red to green colors according to the hierarchical level (red: first level; green: last level). See S4 Data. EEG, electroencephalography. (TIF) [file pbio.3000487.s006.tif]

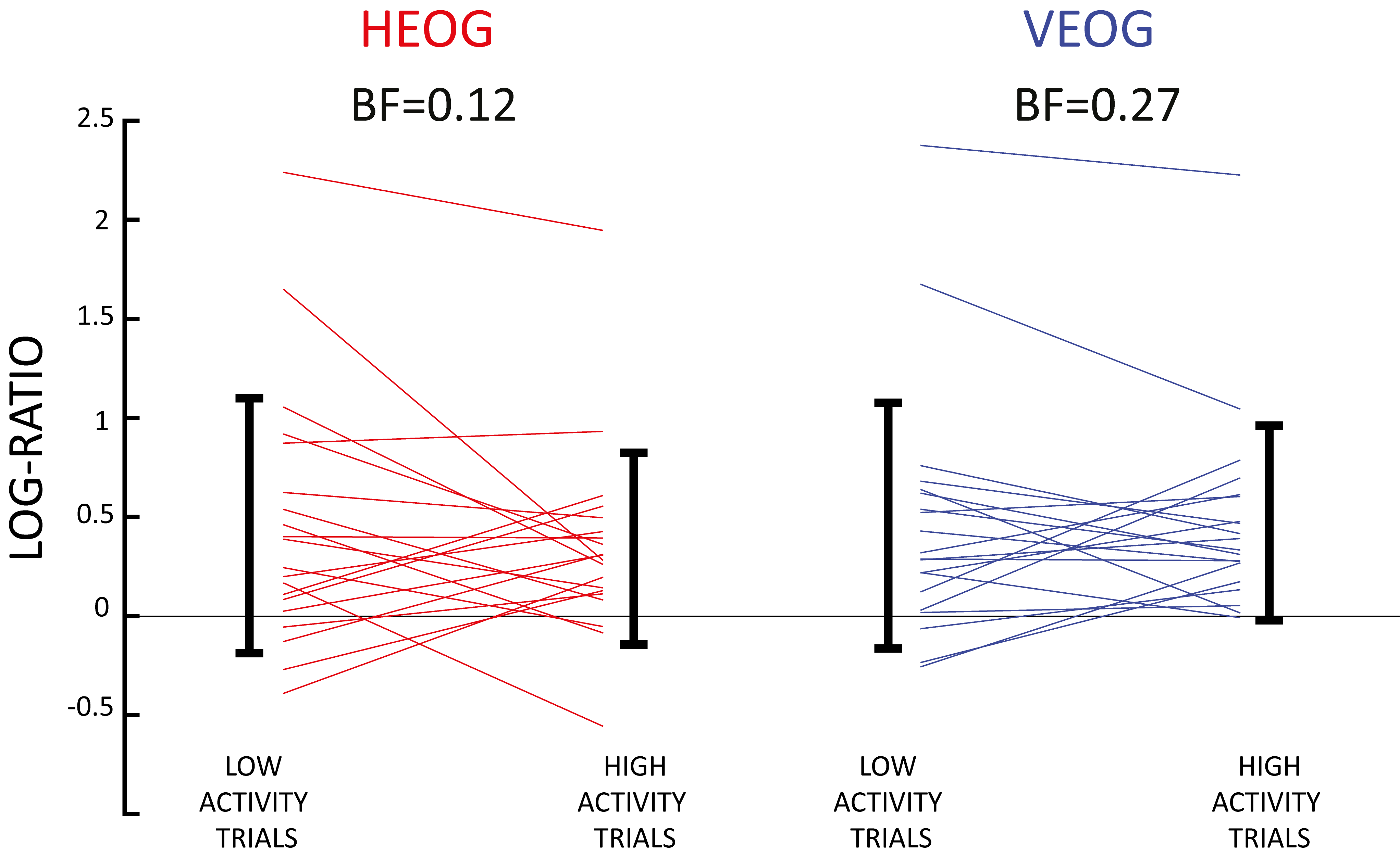

Supplement: S7 Fig — We compared the log ratio between trials with highest versus lowest ocular activity, separately for the HEOG (in red) and VEOG (in blue). The colored lines represent individual participants, with the average ± SE of the mean in black. For both the VEOG and the HEOG, there is no systematic difference in the log ratio, as confirmed by a Bayesian paired-sample t test (both BF < 0.3). The log ratios are overall positive, indicating forward-traveling waves, independent of ocular activity. The HEOG was computed by subtracting the activity in 2 external electrodes set over each participant’s temples. The VEOG was computed by subtracting an external electrode located under the left eye with the signal recorded at the frontal electrode AFz. See S5 Data. BF, Bayes factor; HEOG, horizontal electrooculography; VEOG, vertical electrooculography. (TIF) [file pbio.3000487.s007.tif]

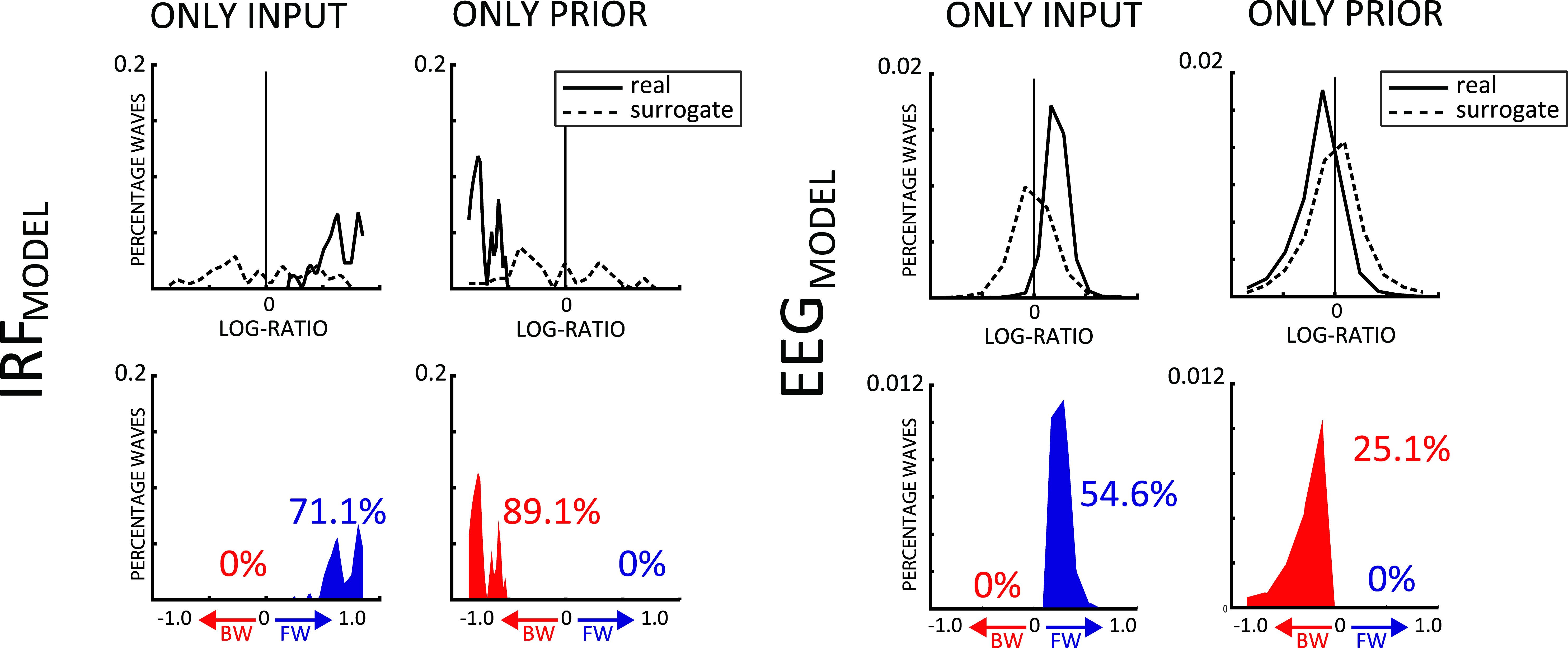

Supplement: S8 Fig — Simulation results when the predictions of every layer are passed through a nonlinear sigmoid function. Similarly to Fig 4 of the main manuscript, the first row shows the real (solid line) and shuffled (dashed line) distributions of log ratios computed over (1) the IRF (2 panels to the left) obtained by cross-correlating predictions with either the sensory inputs (first column) or with the top-down prior signals (second column) and (2) the prediction of the models (considered as a proxy for the EEG signal). The second row shows the (positive values of the) difference between the real and surrogate distributions, representing the traveling-wave events occurring more often than predicted by the null hypothesis. The proportion of significant backward and forward waves are shown in red and blue, respectively. The results of these nonlinear simulations are in line with the ones obtained with the linear system. All data are available at https://osf.io/nc4rg/?view_only=bb06fe996d1f49b285dc25464eec7ac7. EEG, electroencephalography; IRF, impulse response function. (TIF) [file pbio.3000487.s008.tif]

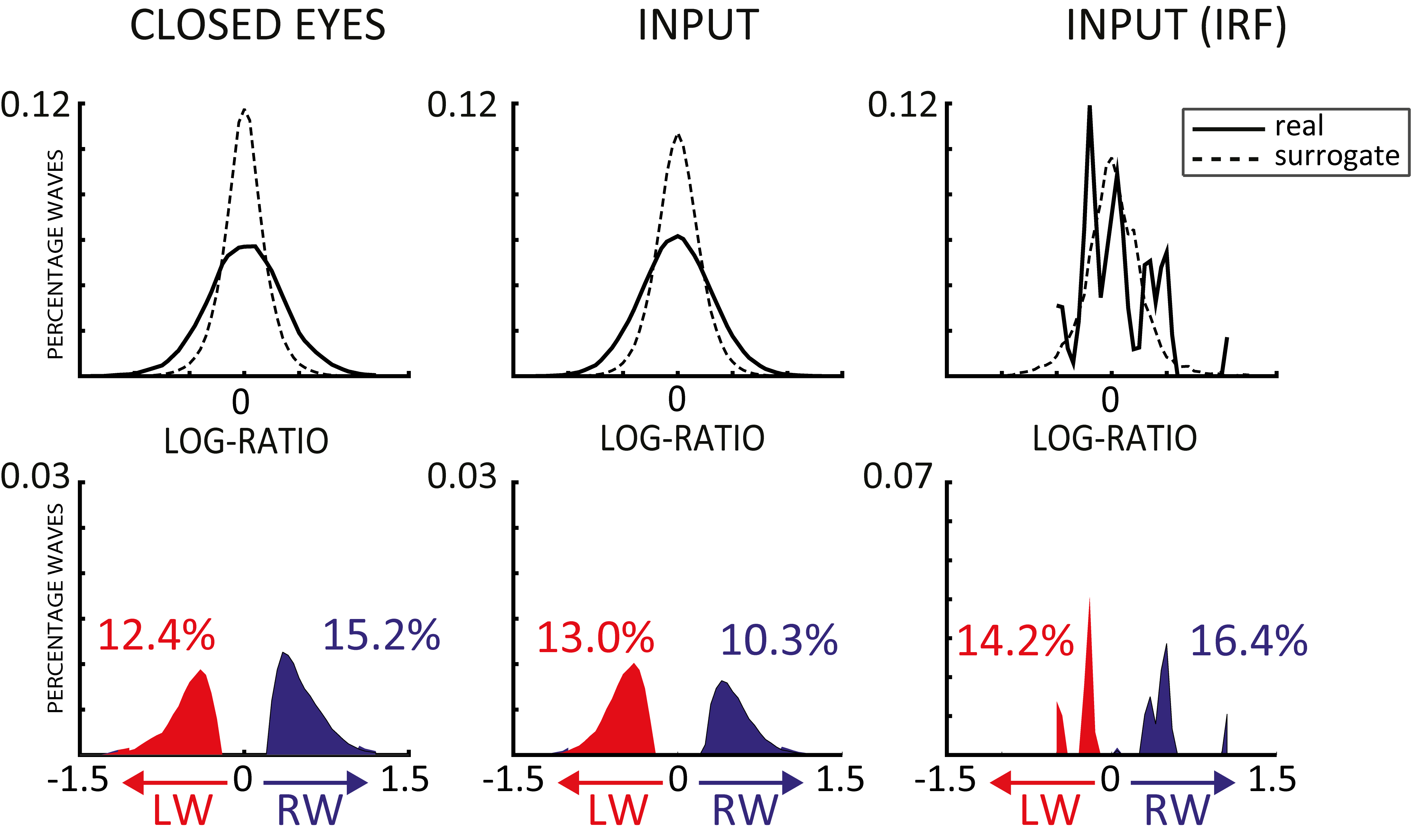

Supplement: S9 Fig — Results for the IRF and EEG from the human EEG datasets (both INPUT and CLOSED EYES) similar to Fig 4B and 4D of the main manuscript, but considering electrodes C5, C3, C1, Cz, C2, C4, C6 (instead of Oz, POz, Pz, PCz, Cz, FCz, Fz). The purpose of this control analysis was to test whether we could detect left-to-right or right-to-left traveling waves. Although the distributions of log-ratio values depart somewhat from the corresponding surrogate distributions (because of the spatial consistency of brain signals in the real data, but not in the surrogate data), there is no preferred direction either for the IRF (rightmost column) or for the raw EEG data (2 leftmost columns). All data are available at https://osf.io/nc4rg/?view_only=bb06fe996d1f49b285dc25464eec7ac7. EEG, electroencephalography; IRF, impulse response function. (TIF) [file pbio.3000487.s009.tif]
